# The Cardiac Pacemaker Story—Fundamental Role of the Na^+^/Ca^2+^ Exchanger in Spontaneous Automaticity

**DOI:** 10.3389/fphar.2020.00516

**Published:** 2020-04-28

**Authors:** Zsófia Kohajda, Axel Loewe, Noémi Tóth, András Varró, Norbert Nagy

**Affiliations:** ^1^MTA-SZTE Research Group of Cardiovascular Pharmacology, Hungarian Academy of Sciences, Szeged, Hungary; ^2^Department of Pharmacology and Pharmacotherapy, Faculty of Medicine, University of Szeged, Szeged, Hungary; ^3^Institute of Biomedical Engineering, Karlsruhe Institute of Technology (KIT), Karlsruhe, Germany

**Keywords:** Na^+^/Ca^2+^ exchanger, pacemaking, sinus node, automaticity, Ca^2+^-handling

## Abstract

The electrophysiological mechanism of the sinus node automaticity was previously considered exclusively regulated by the so-called “funny current”. However, parallel investigations increasingly emphasized the importance of the Ca^2+^-homeostasis and Na^+^/Ca^2+^ exchanger (NCX). Recently, increasing experimental evidence, as well as insight through mechanistic *in silico* modeling demonstrates the crucial role of the exchanger in sinus node pacemaking. NCX had a key role in the exciting story of discovery of sinus node pacemaking mechanisms, which recently settled with a consensus on the coupled-clock mechanism after decades of debate. This review focuses on the role of the Na^+^/Ca^2+^ exchanger from the early results and concepts to recent advances and attempts to give a balanced summary of the characteristics of the local, spontaneous, and rhythmic Ca^2+^ releases, the molecular control of the NCX and its role in the fight-or-flight response. Transgenic animal models and pharmacological manipulation of intracellular Ca^2+^ concentration and/or NCX demonstrate the pivotal function of the exchanger in sinus node automaticity. We also highlight where specific hypotheses regarding NCX function have been derived from computational modeling and require experimental validation. Nonselectivity of NCX inhibitors and the complex interplay of processes involved in Ca^2+^ handling render the design and interpretation of these experiments challenging.

## Introduction

Few areas of cardiac cellular electrophysiology had more intense debate over the years than ‘what makes our hearts beat'—the electrophysiology of the sinus node (SN). In the last two decades, we could witness the struggle of two competing oscillator concepts explaining SN automaticity: in essence, the funny-current (I_f_) and the Ca^2+^-induced depolarization of the membrane potential (V_m_) *via* Na^+^/Ca^2+^ exchanger (NCX, I_NaCa_). Year by year, several studies were published supporting both theories. Thus, the exciting exploration of SN pacemaking mechanisms has been full of debates for over a half century. Numerous mechanisms have been suggested, tested, refuted, accepted, tested, and retested again, updated and after intense point–counterpoint public debates finally settled at a delicate balance known as the coupled-clock mechanism that includes both membrane and sarcoplasmic reticulum (SR) linked contributions. The general field of SN pacemaking (Lakatta et al., 2010; Maltsev et al., 2014; DiFrancesco, 2020) and computational modeling thereof has been excellently reviewed elsewhere (Wilders, 2007; Li et al., 2013). In this review, we focus on the role of NCX function on SN pacemaking and aim to give a balanced view from the very early to recent experimental findings and accompanying computational models.

**Figure 1:**
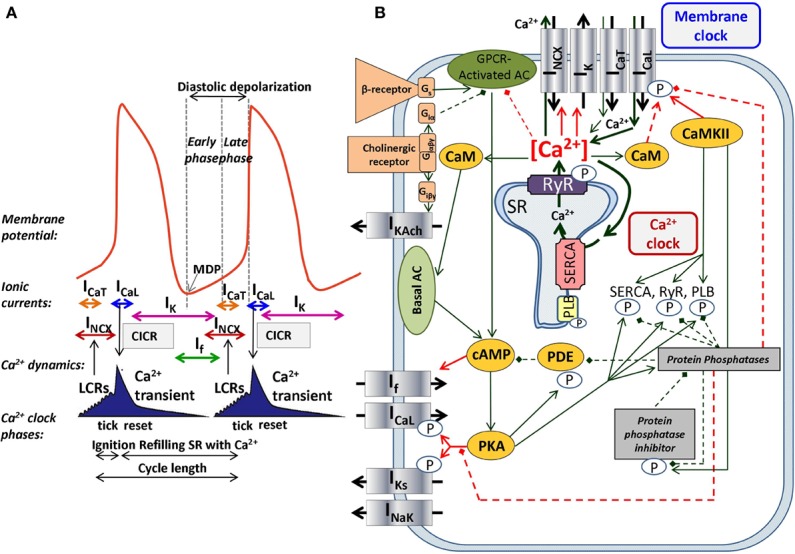
The coupled-clock pacemaker system of the sinoatrial node. Schematic illustration of the currents and functional interactions between the two oscillator subsystem contributing to the SN AP **(A)**. The interplay of the regulatory molecules comprising the coupled-clock pacemaker system is illustrated in **(B)**. Membrane clock (M-clock) of sinoatrial node pacemaker cells comprises the major surface membrane currents: L-type Ca^2+^ channels (I_CaL_), T-type Ca^2+^ channels (I_CaT_), delayed rectifier K^+^ channels (I_K_), hyperpolarization activated funny channels (I_f_), Na^+^/Ca^2+^exchanger (NCX), and Na^+^/K^+^ ATPase (I_NaK_). Regulatory factors that affect the function of proteins of both clocks are indicated with different colors: molecules with red letters couple the Ca^2+^ clock to the M-clock; factors in orange regulate both the M-clock and Ca^2+^ clock *via* the GPCR pathway. Interrupted lines refer to inhibitory effects. AC, adenylate-cyclase; CaM, calmodulin; cAMP, cyclic adenosine monophosphate; CaMKII, Ca^2+^/calmodulin-dependent protein kinase II; GPCR, G-protein coupled receptor; PDE, phosphodiesterase; PKA, protein kinase A; PLB, phospholamban; RyR, ryanodine receptor; SERCA, sarcoplasmic reticulum Ca^2+^ ATPase 2a.

The SNCs show a characteristic slow diastolic depolarization (DD) starting from the maximal diastolic potential (MDP) and ending at the action potential (AP) threshold of about −40 mV. This threshold is called takeoff potential (Kharche et al., 2011). At the takeoff potential, the course of the AP changes qualitatively from slow DD to the AP upstroke (**Figure 1A**). I_f_, NCX, and I_CaT_ (T-type Ca^2+^-current) are mainly active before (at V_m_ more negative than the takeoff potential), whereas I_CaL_ (L-type Ca^2+^-current) and I_Kr_ (rapid delayed rectifier potassium current) are mainly active thereafter (at more positive V_m_), while NCX is active throughout the entire AP cycle. Early studies suggested a major role of a decaying delayed rectifier potassium current (I_Kr_) (Noble, 1962) and the nonselective, cAMP-dependent and hyperpolarization activated current [funny-current, I_f_, (DiFrancesco, 1991)] in the development of DD. This mechanism, governed by transmembrane ion channels with Hodgkin–Huxley kinetics was termed as “membrane-clock” (M-clock). Later studies identified rhythmic, spontaneous locally propagating subsarcolemmal Ca^2+^ releases (LCR) generated by the SR *via* ryanodine receptors (RYRs) during the DD (**Figure 1B**) (Huser et al., 2000; Bogdanov et al., 2001; Monfredi et al., 2013). The LCRs activate the forward mode of the Na^+^/Ca^2+^ exchanger generating inward current that contributes to the DD (Bogdanov et al., 2001). Under certain experimental conditions, and at least temporarily, this mechanism can operate independently of the M-clock, therefore it was termed as “Ca^2+^-clock” (Maltsev et al., 2006). Many of the effects of the LCRs can be represented by their influence on the Ca^2+^ concentrations in the different compartments of the cell as done in most computational models of SNCs. After intense and long debate, several studies provided evidence that the M-clock and Ca^2+^-clock are functionally coupled, converging to the concept of a coupled-clock system (**Figure 1B**). The coupling is based on numerous voltage-, time- and Ca^2+^-dependent mechanisms (Maltsev and Lakatta, 2009; Lakatta et al., 2010) including an important role of the L-type Ca^2+^ current that “resets” and “refuels” the Ca^2+^-clock (Maltsev and Lakatta, 2009) (**Figure 1**). Both I_CaL_ and NCX have important roles in the two clock-like subsystems, *i.e.* they contribute to both the membrane voltage-clock and the Ca^2+^-clock (**Figure 1B**).

## The Role of NCX in the Ca^2+^ Flux Balance

Na^+^/Ca^2+^ exchangers were identified as members of the Ca^2+^/cation antiporters (CaCA) superfamily, as the exchanger carries three Na^+^ in exchange for one Ca^2+^. Thus, the exchanger is electrogenic and affects the transmembrane voltage. The first data regarding the exchange ratio of NCX was published by Reuter and Seitz describing the Na^+^/Ca^2+^ exchange as neutral, *i.e.*, changing two Na^+^ ions for one Ca^2+^ (Reuter and Seitz, 1968). Later, in squid axon experiments, it became clear that the NCX function is electrogenic, with a suggested stoichiometry of 4:1 (Mullins and Requena, 1979). Chapman and Tunstall (1980) suggested a 3:1 ratio in the heart muscle (Chapman and Tunstall, 1980). Nowadays, the 3:1 Na^+^/Ca^2+^ stoichiometry is clearly supported by several experimental studies and generally accepted as the functional exchanger ratio (Bers and Ginsburg, 2007). The electrogenic operation of the Na^+^/Ca^2+^ exchanger results in an ion current with one net elementary charge in each transport cycle. The mode in which the exchanger operates is defined by the relationship of V_m_ and the NCX equilibrium potential (E_NCX_), where E_NCX_ is determined by the transmembrane Na^+^ and Ca^2+^ gradients (E_NCX_ = 3E_Na_ − 2E_Ca_). When the difference between V_m_ and the equilibrium potential is positive, the exchanger acts in the reverse mode; however Ca^2+^ extrusion is favored when V_m_ is more negative than the equilibrium potential (Bers, 2002). Depending on the mode of action (forward *vs.* reverse), the Na^+^/Ca^2+^ exchanger can generate either inward or outward current, it can depolarize or repolarize the cell membrane, respectively.

The activity of the exchanger has complex intra- and extracellular regulatory molecules: Among the *intracellular factors*, the Ca^2+^_i_, ATP, phosphatidylinositol-di-phosphate-2 (PIP_2_), NO and *extracellular factors* such as monovalent cations, PKC activators, and agents that induce proteolysis or protein phosphorylation can activate the exchanger (Ballard and Schaffer, 1996; Bers, 2001; Bers, 2008). Other *intracellular factors* like Na^+^_i_, H^+^, bivalent and trivalent cations, and protein dephosphorylation are inhibitory factors of NCX function (Ballard and Schaffer, 1996; Bers, 2001; Bers, 2008). Modulatory proteins of NCX are PKA, PKC, PP1, and PP2A (Ballard and Schaffer, 1996; Bers, 2001; Bers, 2008). Based on previous experimental results, the modulatory effect of the cAMP-PKA pathway on the NCX function is controversial. Several studies have shown PKA-related activation of the Na^+^/Ca^2+^ exchanger with forskolin and isoproterenol (Iwamoto et al., 1995; Iwamoto et al., 1996; Iwamoto et al., 1998); however, other studies do not demonstrate such effects. Latest studies concluded that the NCX function is not modified by PKA activation (Ginsburg and Bers, 2005).

Three mammalian NCX isoforms have been identified: NCX1 is expressed in the heart, kidney, and brain, having the most extended spectrum of expression among the isoforms, NCX2 is present in the brain, and NCX3 is expressed in the brain and skeletal muscle (Nicoll et al., 1990; Reilly and Shugrue, 1992; Kofuji et al., 1994; Li et al., 1994; Nicoll et al., 1996).

## Pharmacology of the Na^+^/Ca^2+^ Exchanger

Since NCX plays a crucial role under physiological circumstances in the maintenance of Ca^2+^ flux balance and SN pacemaking as well as in pathological settings, *e.g.*, in arrhythmia generation (delayed afterdepolarizations, Ca^2+^ overload), selective inhibition of the exchanger became one of the most intensive research areas demanding the development of properly selective agents. In this chapter, we summarize the NCX inhibitors from the early nonspecific substances to novel, highly selective agents.

### Nonspecific Inhibitory Substances

Divalent (Co^2+^, Sr^2+^, Mg^2+^, Cd^2+^, Ba^2+^, Mn^2+^) and trivalent (La^3+^, Nd^3+^, Tm^3+^, Y^3+^) cations have a nonspecific inhibitory effect on the NCX current (Wakabayashi and Goshima, 1981; Trosper and Philipson, 1983) resulting from direct action on the NCX1 isoform or by replacing Ca^2+^ ions at the transport sites. It is important to note that these cations are nonselective blockers, even the most commonly used Ni^2+^ has nonspecific effects. Ni^2+^ is used in concentrations between 1 and 10 mM and inhibits the reverse mode rather than the forward mode of NCX.

### “Selective” Benzyloxyphenyl Derivative Inhibitors

Some benzyloxyphenyl derivatives such as KB-R7943 (2-[2-[4-(4-nitrobenzyl-oxy)phenyl]ethyl]isothio-urea-methanesulfonate, carbamidothioic acid) were developed as novel NCX inhibitors with promising blocking potency; however experimental studies reported that KB-R7943 exerts complex nonspecific effects on other transmembrane ion currents including I_CaL_, I_Na_, I_Kr_. The IC_50_ values of KB-R7943 are 3.35 ± 0.82 µM on the forward mode and 4.74 ± 0.69 µM on the reverse mode (Birinyi et al., 2005). SEA-0400 (2-(4-(2,5-difluorobenzyloxy)phenoxy)-5-ethoxy aniline) has improved selectivity compared to KB-R7943. However, an I_CaL_ inhibition of approximately 20% was reported (Birinyi et al., 2005). The reverse and forward modes were equally inhibited, having IC_50_ values of 108 ± 18 nM and 111 ± 43 nM, respectively (Birinyi et al., 2005).

### Novel NCX Inhibitors With Increased Selectivity

Due to the lack of selective NCX inhibitors, the exact role of the exchanger in SN pacemaking, cardiac arrhythmogenesis, and Ca^2+^-handling could not be investigated directly. Efforts were made continuously to develop a potent, selective inhibitor to clarify the role of this highly complex transport system in cardiac function. Recently, two novel NCX blocking compounds were identified: ORM-10103 and ORM-10962. The selectivity of both agents was intensively tested by (Jost et al., 2013) and (Kohajda et al., 2016). While ORM-10103 has improved selectivity compared to previous blockers, it still exhibits minor nonspecific effects of inhibiting I_Kr_ at 3 μM. The estimated EC_50_ values of ORM-10103 at 1 μM concentration for the forward and reverse modes of the NCX are 800 and 960 nM, respectively. In contrast, the EC_50_ values of ORM-10962 on the inward and outward NCX current are 55 and 67 nM, respectively. The inhibitory effect of ORM-10962 is highly selective with high efficacy on the NCX current and no effect on the L-type Ca^2+^ current, peak and late sodium current, sodium-potassium pump, and all the repolarizing potassium currents (I_Kr_, I_Ks_, I_to_ (transient outward potassium current), I_K1_ (inward rectifier potassium current)) (Kohajda et al., 2016). The effect of selective NCX-inhibition on SN pacemaking by using 1 μM ORM-10962 was also investigated recently (Kohajda et al., 2020).

### NCX Activators

Probably the most important activating factor for NCX is Ca^2+^ itself. The activation is strongly promoted by elevated Na^+^_i_ and increased by higher pacing frequency (Ginsburg et al., 2013). Pharmacological selective activation of the forward NCX mode is expected to facilitate Ca^2+^ extrusion. The utility of a possible NCX activator compound is controversial, since the facilitation of the forward NCX function could be useful under Ca^2+^ overload; however the arrhythmogenic inward current would also be increased, especially in ventricular cells. A possible increase of the reverse mode, at the same time, could largely modulate this effect. In SNCs, activation of the forward NCX mode may accelerate the firing rate without *β*-adrenergic activation and may decrease intracellular Ca^2+^. However, currently there is no selective NCX activator compound available. Li^+^ is known to stimulate NCX, but its effect on NCX1 is markedly smaller than on NCX2 and NCX3 (Nicoll et al., 1990; Iwamoto et al., 1998). Several papers also claimed that NCX1 is sensitive to redox agents (Reeves et al., 1986) (Amoroso et al., 2000; Santacruz-Toloza et al., 2000). A combination of a reductant (GSH, DTT, Fe^2+^) and an oxidant (Fe^3+^, H_2_O_2_) factor is required to enhance NCX. Diethylpyrocarbonate increased Na^+^-dependent Ca^2+^ uptake (Ottolia et al., 2002). Several peptides (concanavalin-A and insulin) were also shown to activate the exchanger (Gupta et al., 1986; Makino et al., 1988).

## Discovery and Characterization of the Role of NCX in SN Pacemaking

### Pioneer Studies Regarding the Role of NCX in SN Pacemaking

The first experimental results regarding NCX function in SNCs were published by Brown et al. (Brown et al., 1984). They suggested that the slow inward current is composed of two different types of inward currents: a fast component, which they attributed to a channel-gated mechanism (I_Ca,f_) and a slow component: NCX triggered by the release of stored intracellular Ca^2+^ (I_NaCa_). Satoh et al. (Satoh, 1993) provided experimental evidence that caffeine applied in the bath solution at concentrations of 1 to 10 mM caused a frequency decrease and arrhythmias in isolated rabbit cells. This work did not discuss the ionic currents (*e.g.*, NCX) in detail but demonstrated that cellular Ca^2+^ overload was induced during exposure of SNCs to caffeine (Satoh, 1993). However, considering the complex effects of caffeine especially if it is applied in the bath solution makes the interpretation extremely difficult. In the same year, Zhou and Lipsius strengthened the initial suggestions of NCX's role in SN automaticity by using latent pacemaker cells isolated from cat atrium (Zhou and Lipsius, 1993). They demonstrated that 1 µM ryanodine notably decreased the spontaneous pacemaking activity but did not stop automaticity, which led to the conclusion that a ryanodine sensitive component contributes to but is not essential for pacemaking. In line with these results, Rigg and Terrar (1996) found that 2 µM ryanodine and 100 µM CPA applied to guinea pig SNCs substantially decreased the spontaneous firing rate (29 ± 2% and 37 ± 6% respectively). They concluded that both agents markedly reduce the transient amplitude and the diastolic Ca^2+^ levels causing various changes in the Ca^2+^ dependent mechanisms and could affect the contribution of NCX. Similar conclusions were drawn one year later by Li et al. (1997) using ryanodine on rabbit isolated SNCs.

**Figure 2:**
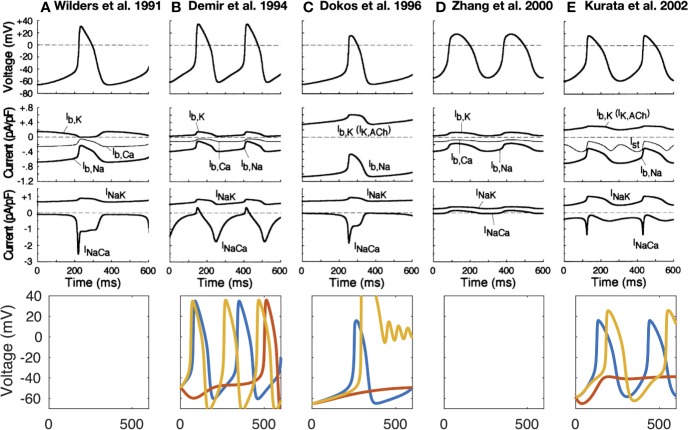
Time-independent background and transporter currents including NCX during spontaneous pacemaking in early SN models: (Wilders et al., 1991) **(A)**, (Demir et al., 1994) **(B)**, (Dokos et al., 1996) **(C)**, (Zhang et al., 2000) [(**D**); central model], and (Kurata et al., 2002) **(E)**. Top 3 rows reproduced from Kurata et al. (2002) with permission. Bottom row: effect of complete NCX block (red line) and clamping intracellular Ca^2+^ to the initial value (yellow line). As the Kurata et al. model comprised a separate subspace Ca^2+^ concentration, this was clamped as well. The Wilders et al. model was not available in the CellML repository, the Zhang model did not exhibit sustained pacemaking behavior in the version deposited in the CellML repository.

The first computational pacemaking model of mammalian SNCs including NCX was proposed by Noble and; Noble (1984) and later extended by Wilders et al. (1991) (Noble and Noble, 1984; Wilders et al., 1991). Another early SN model comprising NCX was proposed by Rasmusson et al. in 1990 (Rasmusson et al., 1990) for bullfrog SNCs. However, in their model parametrization, NCX was small and while modulating the MDP, it had little influence on the AP, which they attributed to Ca^2+^ buffering by myoplasmic proteins. In contrast, the Demir et al. (1994) rabbit model comprised a larger inward NCX (peak −96 pA) during the first third of the pacemaker potential, which then declined slowly through the remainder of the cycle (Demir et al., 1994). The model by Dokos et al. (1996) was mainly driven by an inward background Na^+^ current (Dokos et al., 1996). Upon complete block of their NCX formulation, which reproduced the saturation characteristics at high Ca^2+^_i_ and negative potential low Ca^2+^_i_, automaticity ceased. The Dokos et al. NCX formulation was later on integrated in a rabbit SNC model by Kurata et al. (2002) in which it contributed significantly to DD (Kurata et al., 2002). However, NCX current amplitude decreases during DD in the Kurata et al. model (**Figure 2E**) in contrast to later models introduced below [*e.g.*, (Maltsev and Lakatta, 2009)] where I_NaCa_ increases before I_CaL_ is strongly activated. The NCX time course during the spontaneous APs of different early SNC models is shown in **Figure 2**. For the models available in the CellML repository, **Figure 2** also shows their response to complete NCX block (red lines). The Demir et al. model exhibited CLs as during control after 5 s, *i.e.*, continued to spontaneously generate APs in the absence of NCX (data not shown), the Dokos et al. and Kurata et al. models did not exhibit sustained pacemaking behavior upon complete NCX block. The effect of clamping the intracellular (and in the Kurata et al. model also the subspace) Ca^2+^ concentration to the initial value (*i.e.*, value during early DD) is shown in **Figure 2** as well (yellow lines). Under these conditions, pacemaking ceased in the Dokos et al. model; the CL was shortened in the Demir et al. model, whereas it was prolonged in the Kurata et al. model. **Table 1** compares all SN models comprising a NCX formulation mentioned in this paper. Almost all NCX formulations originate from either DiFrancesco and Noble (1985) or Matsuoka and Hilgemann (1992).

**Table 1:** Comparison of model features for the 21 SNC models described in this review. Only studies that introduced new models or markedly advanced existing models are listed.

Model	Species	NCX formulation	Dynamic intracellular sodium/calcium concentrations	Separate subscarcolemmal space formulation	I_f_	I_Ks_
Noble and Noble, 1984	mammalian	DiFrancesco and Noble, 1985	no/yes	no	yes	no
Rasmusson et al., 1990	bullfrog	Fischmeister and Vassort, 1981	yes/yes	yes	no	no
Wilders et al., 1991	rabbit	Noble and Noble, 1984	yes/yes	no	yes	no
Demir et al., 1994	rabbit	DiFrancesco and Noble, 1985	yes/yes	yes	yes	no
Dokos et al., 1996	mammalian	Matsuoka and Hilgemann, 1992	yes/yes	no	yes	no
Zhang et al., 2000	rabbit	Demir et al., 1994	no/no	no	yes	yes
Kurata et al., 2002	rabbit	Dokos et al., 1996	yes/yes	yes	yes	yes
Garny et al., 2003	rabbit	Zhang et al., 2000	no/no	no	yes	yes
Silva and Rudy, 2003	derived from guinea pig ventricular cells	Luo and Rudy, 1994	yes/yes	no	no	no
Maltsev et al., 2004	rabbit	Kurata et al., 2002	yes/yes	yes	yes	yes
Kurata et al., 2005	derived from human ventricular cells	Priebe and Beuckelmann, 1998	yes/yes	no	no	yes
Maltsev and Lakatta, 2009	rabbit	Kurata et al., 2002	no/yes	yes	yes	no
Chandler et al., 2009	human	Courtemanche et al., 1998	yes/yes	no	yes	yes
Imtiaz et al., 2010	rabbit	Kurata et al., 2002	yes/yes	yes	yes	yes
Kharche et al., 2011	mouse	Kurata et al., 2002	yes/yes	yes	yes	yes
Severi et al., 2012	rabbit	Kurata et al., 2002	yes/yes	yes	yes	yes
Maltsev and Lakatta, 2013	rabbit/human-like	Maltsev and Lakatta, 2009	no/yes	yes	yes	no
Yaniv et al., 2013b	rabbit	Maltsev and Lakatta, 2009	no/yes	yes	yes	no
Stern et al., 2014	rabbit	Maltsev and Lakatta, 2009	no/yes	3D	yes	no
Fabbri et al., 2017	human	Severi et al., 2012	no/yes	yes	yes	yes
Lyashkov et al., 2018	rabbit	Maltsev and Lakatta, 2009	no/yes	yes	yes	yes

In 1998, Ju and Allen (1998) studied the potential role of Ca;^2+^-dynamics in the spontaneous firing rate of APs in sinus venosus of cane toad and provided quantitative data regarding the magnitude of the NCX current during the SN AP. They measured 20–27 pA NCX current in early diastole (Ca^2+^_i_: 250–300 nM) and 12 pA (Ca^2+^_i_: 200 nM) in late diastole in toad isolated sinus venosus cells. The authors emphasized that the net inward current (*i.e.*, total current) required during DD to generate a pacemaker potential is less than 1 pA (DiFrancesco, 1993):

(1) $$I=C_{m}\cdot \frac{dV_{m}\;}{dt}=0.02\frac{V}{s}\cdot 35\;pF=0.7\;pA$$

**Figure 3:**
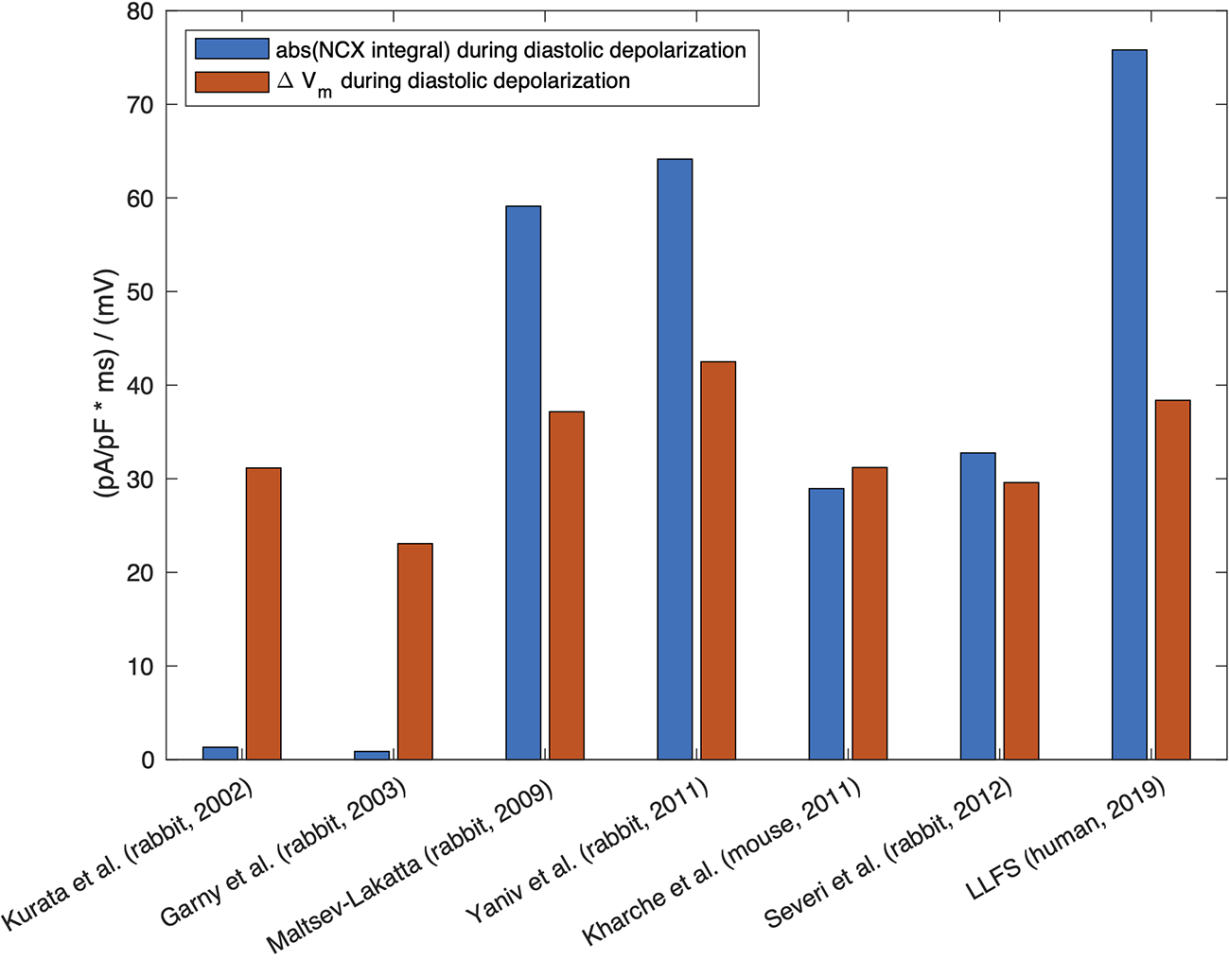
Absolute value of the integral of signed NCX during the DD (MDP to AP takeoff) (blue, unit: ms * pA/pF ≡ mV), in relation to the difference in maximum diastolic V_m_ and takeoff potential for computational models of different species: rabbit (Kurata et al., 2002; Garny et al., 2003; Maltsev and Lakatta, 2009; Yaniv et al., 2011; Severi et al., 2012), mouse (Kharche et al., 2011), and the human Loewe–Lutz–Fabbri–Severi (LLFS) model (Loewe et al., 2019a; Loewe et al., 2019b).

Therefore, quite small relative changes of NCX current (as a response of Ca^2+^_i_ change) can cause substantial changes in the firing rate. Hagiwara and Irisawa (1989) reported 1 pA/pF NCX current density at V_m_ of −40 mV in the presence of 500 nM Ca^2+^_i_ (Hagiwara and Irisawa, 1989). Wilders et al. (1991), in a numerical model, demonstrated that the NCX current had an amplitude of 0.64 pA/pF under similar conditions (Wilders et al., 1991). However, one should keep in mind that the absolute value of NCX current amplitude *per se* does not exclusively define the contribution of the exchanger to DD. The current integral, *i.e.* the total charge transferred through the membrane and its relation to other, concurrently active currents, determines the role for pacemaking, meaning that larger NCX current amplitude does not necessarily imply larger contribution (*cf*. **Figure 3**).

Ju and Allen (1998) also reported that BAPTA and ryanodine (2 µM) caused frequency reduction until the cells stopped beating. However, this happened after 30 min when other nonspecific actions and spontaneous current decline could not be ruled out. They also demonstrated that decrease of external Na^+^ caused a rise of Ca^2+^_i_ due to reverse mode NCX. Application of Na^+^-free solution resulted in a rapid rise in Ca^2+^_i_ that exceeded the preceding systolic Ca^2+^_i_ level. This rapid rise of Ca^2+^_i_ and the generation of an inward current when intracellular [Ca^2+^] is elevated are characteristics of the activation of Na^+^/Ca^2+^ exchange (Allen et al., 1983; Ju and Allen, 1998). The authors also demonstrated that the exchanger current seemed to be close to a linear function of Ca^2+^_i_. *Bufo marinus* pacemaker cells do not contain any I_f_ (Ju et al., 1995). Therefore, I_f_ decrease can be ruled out as a contamination of the experiments. They conclude that NCX has a crucial role in setting the resting heart rate, and the actual level of Ca^2+^_i_ has great importance for indirectly setting the AP firing rate.

### Discovery and Characterization of Local Ca^2+^ Release (LCR) Events: The Role of NCX in SN Automaticity

A seminal study was published by Huser et al. in 2000 (Huser et al., 2000). By using voltage clamp and confocal fluorescence microscopy in cat atrial spontaneously beating cells, they discovered an increase of Ca^2+^_i_ in the last third of DD just before the action potential depolarization, due to local release of Ca^2+^ from the SR. As small dose (25–50 µM) of NiCl_2_ suppressed the local release, thus they suggested the T-type Ca^2+^ channels triggered subsarcolemmal Ca^2+^ sparks, which in turn stimulated the inward NCX to depolarize the pacemaker potential to threshold.

**Figure 4:**
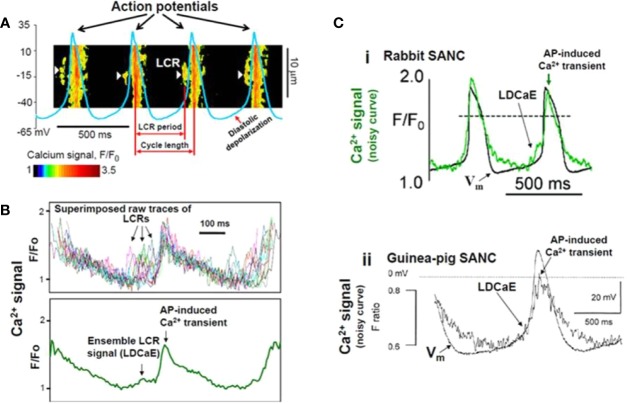
Confocal line scanning image of Ca^2+^-signal with superimposed spontaneous AP traces recorded in rabbit SNCs show DD, distribution of LCRs (white arrows), LCR period, and CL **(A)**. Confocal microscopic image of LCRs are depicted in [(**B**), upper panel], ensemble LCR signal or Late Diastolic Ca^2+^ Elevation (LDCaE) that precedes Ca^2+^-transient are created from temporal average of LCRs [(**B**), lower panel]. LDCaE in single SNC of rabbit [(**C**), panel i] and guinea-pig [(**C**), panel ii] is shown. LCR: local Ca^2+^ release. From (Maltsev et al., 2014) with permission.

One year later, the Lakatta group (Bogdanov et al., 2001) further analyzed the spontaneous Ca^2+^ releases (local Ca^2+^ releases, LCR, **Figure 4**) in isolated rabbit sinoatrial cells. Their findings indicate that the pre-AP releases are locally propagating Ca^2+^ waves, resulting from ryanodine-sensitive Ca^2+^-induced Ca^2+^ release (CICR). In turn, the negative chronotropic effect of ryanodine is the result of disappearance of localized pre-AP Ca^2+^ releases. Substituting Na^+^ for Li^+^ to inhibit NCX caused sinus arrest leading to the conclusion that Ca^2+^ release-NCX cross-talk has crucial importance in pacemaking. The authors provided a possible scenario for the current interactions underlying spontaneous automaticity: after reaching the maximal diastolic potential, I_f_ activates to depolarize the membrane and activates I_CaT_ and I_CaL_ causing LCR from the SR, which in turn activates NCX and the inward current augments to activate the remaining L-type Ca^2+^ channels. These results were interpreted such that SR Ca^2+^ release has a pivotal impact in defining the actual firing rate and inactivation of either the RyR or NCX activity lead to frequency slowing or abolishment.

The pivotal role of the strong interaction between the Ca^2+^ concentration in the subspace and V_m_ *via* NCX for spontaneous depolarizations in SNCs was numerically reproduced and mechanistically underpinned by Maltsev et al. (2004) in a biophysical modeling study in 2004 based on an extension of the Kurata et al. (2002) model (Kurata et al., 2002). In 2009, Maltsev and Lakatta proposed a coupled oscillator model incorporating aspects of both a membrane and a Ca^2+^ clock, which yielded rhythmic spontaneous depolarization over a wider range of parameters of the currents involved in the M-clock (I_CaL_, I_f_) (Maltsev and Lakatta, 2009). By modulating SR Ca^2+^ pumping rate, the coupled-clock yielded robust pacemaking in the range of 1.8 to 4.6 Hz, which is wider than could be achieved by modulation of M-clock properties alone. Kurata et al. (2012) performed stability and bifurcation analyses on this coupled-clock model and found that the most important model parameters for generation of cyclic depolarization (equilibrium point instability) are the SR Ca^2+^ pumping rate, NCX density as well as I_CaL_ conductance (Kurata et al., 2012). However, the model system does not exhibit cyclic intracellular Ca^2+^ oscillations when V_m_ is clamped (independent of the clamped value), *i.e.*, arguing against an independent Ca^2+^ clock, thus underlining the coupled nature of the pacemaking mechanism. In line with this finding, Maltsev and Lakatta (2009) observed damped oscillations when clamping the transmembrane voltage to −65 mV (at P_up_ = 20 mM/s) (Maltsev and Lakatta, 2009). However, sustained oscillations were observed at P_up_ = 40 mM/s. The NCX was a major contributor to the observed equilibrium point in the study by Kurata et al. (2012).

Vinogradova et al. explored in 2004 whether the LCRs require previous membrane depolarization as was suggested so far (Vinogradova et al., 2004). Experimental measurements on isolated rabbit SNCs as well as numerical modeling revealed three findings i) the LCRs during the DD do not require previous membrane depolarization; ii) the LCRs are rhythmic and generate rhythmic inward current movements; iii) the LCR periods are highly correlated with the spontaneous CLs.

In contrast to the increasing body of evidence, Sanders et al. claimed in 2006 that the contribution of NCX to spontaneous depolarization does not exclusively depend on the intact SR function in guinea pig SNCs (Sanders et al., 2006). They demonstrated that low Na^+^ solution abolished spontaneous firing in the presence of CPA, an inhibitor of SR uptake. On the other hand, if NCX would be entirely a consequence of SR Ca^2+^ release, the CPA should have a similar effect as a low Na^+^ solution. However, the SR inhibition only slowed the heart rate, while low Na^+^ abolished spontaneous pacemaking within one beat, with concomitant decrease of Ca^2+^_i_. However, considering the rather complex effects of reduced Na^+^ solution, the results could not unequivocally support the authors' hypothesis. The application of KB-R7943 to inhibit NCX has to be considered problematic because of its nonselectivity.

Using high-speed Ca^2+^ imaging, Yaniv et al. (2013) could show that the coupled clocks also control beat-to-beat regulation of the spontaneous beating rate (Yaniv et al., 2013b). Acute application of low concentrations of caffeine (2 to 4 mM) caused Ca^2+^ release from the SR and subsequently led to increased spontaneous beating rates *via* membrane depolarization caused by increased NCX activation. This effect could be reproduced mechanistically in an extended version of the Maltsev–Lakatta computational model (Yaniv et al., 2013b). Toward this end, Severi et al. (2012) proposed an update of the Maltsev–Lakatta rabbit SNC model incorporating a coupled-clock with Ca^2+^-handling (including NCX) and refined current formulations based on more recent experimental data (Severi et al., 2012). On the other hand, the Severi et al. model could not predict the effect of acute caffeine injection described above and complete block of I_f_ lead to cessation of spontaneous activity. Simulations including the effects of 1 µM isoproterenol [causing, among other, a 25% increase of SR Ca^2+^ pumping as in Maltsev and Lakatta (2010) (Maltsev and Lakatta, 2010)], yielded a 28.2% rate increase in the Severi et al. model in good agreement with the experimentally observed increase of 26.3 ± 5.4% by Bucchi et al. (2007b).

In 2013, Sirenko et al. confirmed the presence of LCR in SNCs (Sirenko et al., 2013). The authors compared the spontaneous Ca^2+^ releases in permeabilized (*i.e.*, in the absence of functional ion channels) rabbit SN *vs.* ventricular cells. They found that in the presence of similar physiological Ca^2+^ concentrations, the SNCs generate large rhythmic spontaneous Ca^2+^ releases in contrast to ventricular cells in which they found Ca^2+^ sparks with small amplitude and stochastic nature, as was originally described (Cheng et al., 1993). This ability of SNCs was associated with more abundant SERCA, reduced extent of phospholamban (PLB), and increased Ca^2+^-dependent phosphorylation of PLB and RyR.

In 2013, Yaniv et al. found new evidence regarding the membrane and Ca^2+^ clock coupling (Yaniv et al., 2013a). Application of the I_f_-inhibitor ivabradine (3 µM) reduced the AP firing rate, partially by inhibiting I_f_ and by decreasing the SR Ca^2+^ content in isolated SNCs. This means that despite the fact that ivabradine has no direct effect on intracellular Ca^2+^ cycling and does not exert effects on surface membrane channels other than I_f_, it still suppresses the SR Ca^2+^ load and the LCR events. These novel findings further supported the hypothesis that cross-talk between the membrane and Ca^2+^ clock regulates SN automaticity.

Sirenko et al. (2016) analyzed in detail the electrochemical Na^+^/Ca^2+^ gradients in the pacemaker function of the NCX (Sirenko et al., 2016). They used a combination of numerical modeling and experimental rabbit approaches (by using the Na/K inhibitor digoxigenin) to determine how the coupling of Na^+^ and Ca^2+^ electrochemical gradients regulates pacemaking. Minimal increase of Na^+^_i_ (5%) enhanced the LCRs, shortened the CL, which was interpreted as an extension of normal chronotropic response. Further increase of Na^+^_i_ by up to 15 mM caused reduction of LCRs, large CL variability as a consequence of V_m_-Ca^2+^ clock uncoupling. In parallel with the biphasic CL changes, the NCX current also showed biphasic alterations: initially the I_NCX_ increased due to the high amplitude LCRs, then declined as E_NCX_ reduced. The authors claimed that E_Na_, E_Ca_, and E_NCX_ tightly regulate the SN automaticity *via* influencing several ion channels and components of the SN clocks.

Stern et al. (2014) proposed a computational model with subcellular spatial resolution that allows studying intracellular Ca^2+^ release propagation patterns that was employed in Maltsev et al. (2017) to classify LCRs and provide insight into mechanisms increasing robustness of pacemaking at various SERCA pumping rates. Consistent with previous studies, they found a major role of NCX in the fight-or-flight response: faster SR Ca^2+^ pumping prepared the cell for stronger (higher amplitude) and more synchronous LCRs (more subcellular Ca^2+^ wave collisions) in the next cycle, which elicited a higher NCX current.

Torrente et al. in 2016 studied the role of the predominant isoform of L-type Ca^2+^ channels (Ca_v_1.3) in the generation of the LCRs and Ca^2+^_i_ dynamics in SNCs from wild-type and Ca_v_1.3 knockout mice (Torrente et al., 2016). This work challenged the results of previous experimental work (under voltage-clamp conditions with V_m_ below the activation of Ca_v_1.3 (Vinogradova et al., 2004) and in membrane-permeabilized SNCs, without the influence of any plasma membrane ion channels (Sirenko et al., 2013) claiming that LCRs are clearly spontaneous in nature and entirely independent from the M-clock. Ca_v_1.3 deficiency significantly impaired Ca^2+^_i_ dynamics by reducing the frequency of LCR events and preventing synchronization. The results of Torrente et al. suggest that the local increase of Ca^2+^_i_ generated by Ca_v_1.3 Ca^2+^ current induces RyR opening, thus supporting the coupling between membrane depolarization and SR Ca^2+^ release. Thus Ca_v_1.3 channels can be inducers of LCR generation in the late DD. Considering this mechanism, Ca_v_1.3 could stimulate the NCX-mediated depolarizing current to reach the threshold potential necessary to trigger an AP. Although LCRs are likely triggered by Ca_v_1.3-mediated I_CaL_ release in mouse SNCs (Torrente et al., 2016), LCR generation has different mechanisms in pacemaker cells of various species. As mentioned before, Vinogradova et al. showed that LCRs in rabbit SNCs do not require a change of V_m_ (Vinogradova et al., 2004). Instead, LCRs are spontaneous in nature and occur in permeabilized SNCs. Takimoto et al. showed that the *α*_1D_ Ca^2+^ channel mRNA is expressed in rat atrium (Takimoto et al., 1997). This observation was confirmed in mouse and human cells by Mangoni et al. (Mangoni et al., 2003). However, it remains unclear whether *α*_1D_ Ca^2+^ channels are present and functional in rabbit SNCs since expression at the protein level has not been verified (Qu et al., 2005).

Huser et al. found that T-type Ca^2+^ channels can also have a role in the generation of LCRs since in cat atrial pacemaker cells, LCRs are triggered by voltage-dependent activation of T-type Ca^2+^ channels (Huser et al., 2000).

### The Role of Phosphorylation in SN Pacemaking and the Fight-or-Flight Response

A crucial feature of the SN is the capability of adapting the firing rate to the momentary requirements of the body. *β*-adrenergic activation stimulates the cAMP-PKA cascade and phosphorylates several components of the Ca^2+^ handling machinery and transmembrane ion channels. As a consequence, higher frequency and magnitude of LCRs provide enhanced drive for NCX, thus accelerating DD (Vinogradova et al., 2005; Vinogradova and Lakatta, 2009).

#### CaMKII

In 2000, Vinogradova et al. reported a crucial role of CaMKII in SN pacemaking (Vinogradova et al., 2000). Selective inhibition of CaMKII activity (by autocamtide 2-inhibitory peptide, and KN-93/KN-92) reduced the pacemaker frequency in rabbit SNCs, furthermore, larger doses of the compounds completely terminated SN automaticity. The authors also demonstrated that CaMKII activity is driven by subsarcolemmal Ca^2+^ movements and located in the vicinity of L-type Ca^2+^ channels. Therefore, the vital role of CaMKII could be based on the modulation of I_CaL_ *via* subsarcolemmal Ca^2+^ changes (Vinogradova et al., 2000).

In 2011, Gao and Anderson showed that CaMKII can support heart rate increase independent of *β*-adrenergic stimulation (Gao et al., 2011). Furthermore, CaMKII is required for *β*-adrenergic response and Ca^2+^-based mechanism of pacemaking.

Li et al. demonstrated that CaMKII inhibition reduced phosphorylation of RyR and PLB, decreased the LCR size, and increased the LCR period (Li et al., 2016). As a consequence, SN firing rate was reduced. These results led the authors to the conclusion that high basal CaMKII activation ultimately regulates the pacemaker function *via* phosphorylation of Ca^2+^ handling proteins.

#### cAMP-PKA-PDE

The Lakatta group in 2008 demonstrated considerably high basal activity of phosphodiesterase (PDE) enzyme, which restricts local RyR Ca^2+^ release during DD *via* reduction of cAMP PKA-dependent protein phosphorylation to keep the basal spontaneous SNC firing under control (Vinogradova et al., 2008). Also in 2008, Younes et al. demonstrated in isolated SNCs that the adenylate-cyclase is activated in the entire physiological concentration range of intracellular Ca^2+^ and the cAMP-PKA axis activation drives SR Ca^2+^ release, which in turn activates AC (Younes et al., 2008). This feed-forward “fail-safe” system has a crucial role in maintaining the normal rhythm of SNCs.

In 2011, Liu et al. studied the role of the AC-cAMP-PKA-Ca^2+^ signaling cascade in mouse SNCs, and they demonstrated that the Ca^2+^ handling proteins are abundantly expressed, and LCRs were also detected in skinned cells. They showed that inhibition of intrinsic PKA activity reduces PLB phosphorylation and prolongs the LCR period causing reduction in the SNCs firing rate. The PDE inhibition resulted in the opposite effects: increased PLB phosphorylation, shortened LCR period, and accelerated firing. In the same study the authors also demonstrated that *β*-adrenergic activation requires intact Ca^2+^ handling, indicating a pivotal role of AC-cAMP-PKA-Ca^2+^ signaling cascade in maintaining the normal automaticity in mouse SNCs (Liu et al., 2011).

Vinogradova et al. provided data regarding the synergistic role of PDE3/PDE4 activity in rabbit SNCs in 2018. Individual inhibition of PDE3 or PDE4 caused a moderate increase in the beating rate (20 *vs.* 5%, respectively) (Vinogradova et al., 2018a). However, parallel block of both caused a 45% increase of pacemaking rate, indicating a synergistic effect. Furthermore, dual inhibition enhanced the LCR numbers and size, reduced the SR refilling time and LCR period due to decrease of cAMP/PKA phosphorylation (Vinogradova et al., 2018b). An amplification of local RyR Ca^2+^ release activates augmented NCX current at earlier times leading to an increase in the DD rate and spontaneous SANC beating rate. When RyRs were disabled by ryanodine, PDE inhibition failed to amplify local Ca^2+^ releases and increase NCX current. As a result, there was no increase in the DDR and spontaneous SN beating rate (Vinogradova et al., 2018b). In this context, Vinogradova et al. showed in the same year that PDE3/PDE4 modulation of SNCs is self-adaptive, *i.e.*, full functional effect is achieved only when both PDEs are inhibited. Such inhibition will lead to an elevation of the local level of cAMP and PKA phosphorylation. The authors claimed that local cAMP signals may have greater importance in the regulation of spontaneous automaticity in SNCs than the ‘global cAMP' (Vinogradova et al., 2018a).

#### Autonomic Modulation

In 2002, Vinogradova et al. elucidated the Ca^2+^ release during DD as the specific link between *β*-adrenergic stimulation and increased firing rate in rabbit SNCs (Vinogradova et al., 2002). They demonstrated that 3 µM ryanodine abolished the effect of 0.1 µM isoproterenol. In the presence of ryanodine, the *β*-adrenergic stimulation failed to alter the slope of DD, firing rate, and subsarcolemmal Ca^2+^ releases, whereas the I_CaL_ amplitude exerted clear *β*-adrenergic stimulation-induced increase. In 2006, Bogdanov et al. described how application of agents affecting the timing or amplitude of LCRs (ryanodine, BAPTA, nifedipine or isoproterenol) caused immediate changes of spontaneous beating rate, which could be traced back to changes of local NCX using mechanistic computational modeling (Bogdanov et al., 2006). Similarly, PKA-dependent phosphorylation affected LCR spatiotemporal synchronization and therefore modulated beating rate mediated by altered NCX (Vinogradova et al., 2006).

Himeno et al. (2008) studied *β*1-adrenergic stimulation in a computational model of guinea pig SNCs with large I_Ks_ in which they described the role of NCX (Himeno et al., 2008). While activation of I_Ks_ during the AP preceding the *β*1-adrenergic stimulation was negligibly small, I_Ks_ counterbalanced the increase in I_CaL_ and NCX, which otherwise compromised the positive chronotropic effect of the *β*1-adrenergic stimulation by elongating the APD in their model.

Lyashkov et al. (2009) investigated the mechanism of muscarinic-receptor stimulation on heart rate reduction (Lyashkov et al., 2009). They found that carbachol (at IC_50_) reduced the number and size of LCRs and lengthened the LCR period with concomitant decrease of the beating rate. Numerical modeling indicated that cholinergic-modulation of the beating rate is integrated into the Ca^2+^ cycling *via* LCR-mediated function of the NCX. The authors concluded that in the presence of low doses of carbachol, the muscarinic activation induced beating rate reduction is mediated by suppression of cAMP-PKA-Ca^2+^ signaling, while I_K(Ach)_ activation contributes only under higher carbachol concentrations.

In the same year, Maltsev and Lakatta (2010) published a modeling study demonstrating how G protein-coupled receptor modulation of spontaneous SNC beating rate is not only mediated by effects on membrane currents but also by effects on the SR Ca^2+^ pumping rate, which in turn affect diastolic NCX (Maltsev and Lakatta, 2010). Also in 2010, Gao et al. demonstrated in healthy canine hearts that complete or almost complete I_f_ block attenuated but did not eliminate the positive chronotropy of isoproterenol suggesting that I_f_ is not the only target of the *β*-adrenergic response (Gao et al., 2010). Their results also support the notion of spontaneous Ca^2+^ releases during late DD, which was sensitive to isoproterenol. In their experiments, 2 µM ryanodine caused a 14% decrease in pacemaker frequency but dramatically decreased the effect of isoproterenol. They also provided evidence that ryanodine has no direct effect on I_f_. The authors concluded that both voltage and Ca^2+^ dynamics have a role in the pacemaker mechanism.

In the same year, Vinogradova and Lakatta demonstrated that the LCR period and the spontaneous SN cycle is tightly regulated by the SR refilling time in rabbit SNCs (Vinogradova et al., 2010). Therefore, phosphorylation/dephosphorylation of the SERCA regulator protein PLB has also critical roles in setting the actual CL of the SN automaticity especially during *β*-adrenergic stimulation.

In a theoretical analysis of the relative importance of the membrane and Ca^2+^ oscillators, Imtiaz et al. (2010) could show that increased SR Ca^2+^ uptake leads to i) hyperpolarized MDP *via* a reduction of NCX due to lower Ca^2+^_i_ (Imtiaz et al., 2010). This means that due to lower Ca^2+^ the smaller inward current through NCX enables more negative MDP. ii) The more negative V_m_ increases the magnitude of I_f_ and I_CaT_ during early diastole. iii) The enhanced Ca^2+^ entry during DD increases intracellular Ca^2+^ and CICR causing a higher NCX current.

### Transgenic Modifications of NCX

In 2013, several studies using transgenic NCX knockout mice were published. First, Gao et al. used cardiac specific, *incomplete* NCX1 knockout mice where the baseline sinus frequency was completely identical between control and NCX1-deleted mutants (Gao et al., 2013). However, they found that NCX1 deletion only partially impaired the isoproterenol effect indicating that I_f_ is also required. The authors concluded that the NCX has a minor role in maintaining the normal heart rate but NCX1 deletion dramatically enhanced the effects of ivabradine on isoproterenol-induced automaticity indicating that NCX and I_f_ mutually contribute in the fight-or-flight response. Since the genetic knockout of NCX was incomplete, one cannot rule out the possibility that the remaining NCX may be able to provide enough current to fulfill its role in maintaining the Ca^2+^ balance.

In response to that paper, Maltsev et al. in 2013 provided rabbit SN numerical simulations (Maltsev et al., 2013) to address some unanswered questions by Gao et al. (2013). The authors extended their simulation with new stabilization of NCX *via* local control of CICR. They argued that the Ca^2+^ released from the RyR is able to recruit the neighboring RyRs producing Ca^2+^ wavelets (LCRs) having amplitudes larger than the Ca^2+^ sparks and smaller than global Ca^2+^ transients. Since the NCX extrudes Ca^2+^ from the vicinity of RyRs it attenuates the spread of Ca^2+^ leading to weaker recruitment. However, when NCX is genetically decreased (*i.e.* incomplete NCX1 knockout), the Ca^2+^ spread and recruitment are enhanced providing larger Ca^2+^ and driving force for the remaining NCX molecules. These results indicate that NCX has a crucial role under the normal heart rate, and a small fraction (*i.e.* 20%) of NCX is able to produce sufficient current under DD. However, when NCX expression is low, its capacity is fully used for Ca^2+^ extrusion under rest and there is no more support for frequency increase under *β*-adrenergic response.

Herrmann et al. in 2013 used inducible and SN specific Cre transgenic mice lacking NCX1 selectively in SN pacemaking cells (Herrmann et al., 2013). The NCX1 was genetically ablated in a temporally controlled and tissue selective manner. The animals exerted severe bradycardia and large variability with arrhythmias leading to the conclusion that NCX has a role to maintain normal rhythm. The caffeine induced transient decay was markedly slowed (3.6% of control) in cpNCXKO cells indicating that in SNCs, almost the entire trans-sarcolemmal Ca^2+^ extrusion is carried by NCX. At the same time, it is an interesting finding that the Ca^2+^ transient amplitude was decreased and the decay was slowed while the SR Ca^2+^ content was unaffected. The authors claimed that the NCX1 deleted cells are not able to compensate the NCX1 deletion. They argue for a fundamental role of NCX in pacemaking even under resting conditions, and the normal pacemaking function is not possible without proper NCX function.

Groenke et al. found that in NCX1 knockout mice (Cre/LoxP system), there is no indication of spontaneous atrial depolarizations on the ECG and the animals exerted junctional escape rhythm (Groenke et al., 2013). Furthermore, isolated cells did not show spontaneous automaticity despite the presence of I_f_ and intact Ca^2+^ stores. They claim that NCX has a crucial role in normal pacemaking. They observed LCRs but no Ca^2+^ transients in NCX deleted cells. They also conclude that I_f_ is not sufficient to depolarize the cell membrane if NCX is completely lacking, interestingly not even if isoproterenol was added. Torrente et al. in 2015 created an atrial-specific NCX knockout mouse where NCX was totally eliminated from the atria including the SN (Torrente et al., 2015). These cells lacked spontaneous APs despite intact I_f_. In contrast, the intact tissue showed spontaneous burst–pause type depolarizations. The authors conclude that in the absence of NCX-mediated depolarizations, I_CaL_ and I_f_ are able to initiate AP burst where Ca^2+^ is gradually accumulated and finally terminated burst activity *via* Ca^2+^-dependent inactivation or by other Ca^2+^ activated processes such as small-conductance Ca^2+^-activated K^+^ currents (Torrente et al., 2017).

In 2016, Choi et al. observed regular oscillations of inward currents in voltage-clamped human embryonic stem cell-derived cardiomyocytes, which exhibit spontaneous APs when not being voltage clamped (Choi et al., 2016). The oscillatory Ca^2+^ releases could be eliminated by blocking NCX (with 1 µM SN-6 and 10 µM KB-R7943) but not by blocking I_f_ (with 3 mM ivabradine). Their observations suggest that in these hESC-CMs, the M-clock and the voltage clock act as two redundant pacemaker mechanisms which can work independently.

Kaese et al. (2017) used a homozygously overexpressed NCX mouse model and found that the basal heart rate is not affected, suggesting that NCX is not a crucial factor in setting the resting heart rate (Kaese et al., 2017). In contrast, *β*-adrenergic stimulation elicited higher heart rates in NCX-overexpressing mice, suggesting more inward NCX current which enhances the speed of DD.

Between 2003 and 2011 several genetically manipulated mice studies with deficient I_f_ function were published where autonomic rate modulation was preserved (Ludwig et al., 2003; Herrmann et al., 2007; Harzheim et al., 2008; Hoesl et al., 2008; Baruscotti et al., 2011). These results further indicate the lack of an essential and irreplaceable role of I_f_ in SN pacemaking and emphasize that I_f_ works together with its “teammates” within the coupled-clock system (Lakatta and Maltsev, 2012)

### AP Ignition Model as the Most Current SN Pacemaking Mechanism

In 2018, a new model was proposed by the Lakatta group extending SN electrophysiology by an integrated regulatory mechanism including close interactions of LCRs, NCX, and I_CaL_ coupled by a positive feedback *via* diastolic CICR and DD acceleration (Lyashkov et al., 2018). In contrast to previous theories arguing for a role of LCRs during the late DD, the AP ignition model suggests that early LCRs (I_CaL_-independent) activate inward NCX, which defines the ignition onset. I_f_, and I_CaT_ also contribute to early depolarization to reach the I_CaL_ threshold. The physiological role of the low-voltage activation threshold L-type Ca^2+^ channel isoform (Ca_v_1.3) may have more importance to regulate the early ignition phase. However, the details of the specific contribution of Ca_v_1.3 require further investigation. The I_CaL_-mediated Ca^2+^-influx recruits more LCRs *via* diastolic CICR, and this positive feedback further increases NCX. It was also shown that I_CaT_ also has a role in the diastolic CICR, similarly as early results suggested (Huser et al., 2000). However, previously ivabradine and ryanodine were considered to cause AP CL lengthening by different mechanisms (*i.e.* ivabradine by I_f_ inhibition and ryanodine by takeoff potential depolarization). The underlying mechanism of ryanodine induced decrease in the firing rate could be driven by the marked decrease in DDR, which was observed in isolated rabbit sinoatrial node cells by Vinogradova et al. (2002). The AP ignition model brings them common ground: regardless of whether the M-clock (in case of ivabradine) or the Ca^2+^-clock (in case of ryanodine) is perturbed, it will indirectly affect the other clock *via* the coupling mechanism. As a coupling, similar responses happened, the ignition potential become depolarized, and time-to-ignition phase becomes longer. This provides delay in clock-coupling by CICR delay.

### The Role of NCX in Human SNCs in Comparison to Other Species

In contrast to the significant amount of experimental data on the role of NCX in pacemaking derived from animal experiments (mostly isolated rabbit SNCs), data from human SNCs or tissue are scarce. Considering the vast difference in the main quantity of interest, the beating rate, between commonly used laboratory animals and humans (mouse: 500 bpm, rabbit: 300 bpm, dog: 100 bpm, human: 60 bpm), it however cannot be assumed that the details of pacemaking mechanisms and most importantly the delicate balance of competing effects can be transferred from animal models to human in general. Indeed, it has been shown that computational models of SNCs of different species (mouse, rabbit, human) react markedly different to changes of *e.g.* extracellular Ca^2+^ concentration (Loewe et al., 2019b). Given the potential relevance of this alteration regarding sudden bradycardic death in dialysis patients (Loewe et al., 2019a), species-dependent investigations are desirable. As a particular example of interspecies differences, I_Kr_ has been reported to be absent in porcine SNCs and IKs to be negligibly small in rabbit SNCs during rest, while both are present in guinea pig SNCs (Satoh, 2003). Moreover, SN AP duration is markedly different between species (mouse: 80 ms, rabbit: 200 ms, human: 300 ms; approximate values) as are other electrophysiological parameters such as AP upstroke velocity, MDP, and DDR (Opthof, 2001). Regarding the role of NCX, its importance in terms of relative contribution to Ca^2+^ extrusion differs between species as well: 28% in rabbit compared to 7% in rat, *i.e.*, a 4-fold difference (Bers, 2002).

One of the few experiments using human SNCs was performed by Tsutsui et al. (2018). The authors show that similarly to animal models, spontaneous rhythmic LCRs generated by the Ca^2+^ clock are coupled to electrogenic surface membrane molecules to initiate rhythmic APs, and that Ca^2+^-cAMP-protein kinase A (PKA) signaling has a regulatory role in the clock coupling in human SNCs (Tsutsui et al., 2018). In the absence of strong coupling between the oscillator subsystems, it was shown that SNCs fail to trigger spontaneous APs and only exhibit disorganized LCRs that were unable to activate the M-clock (Tsutsui et al., 2018). In summary, the study by Tsutsui et al. provides evidence that also in humans it is the coupled-clock mechanism that fundamentally drives SNC pacemaking and provides hints for mechanisms underlying SN dysfunction (Tsutsui et al., 2018).

To highlight interspecies differences and provide an overview of the characteristics of the role of NCX in different models, basic *in silico* experiments were performed.

**Figure 5:**
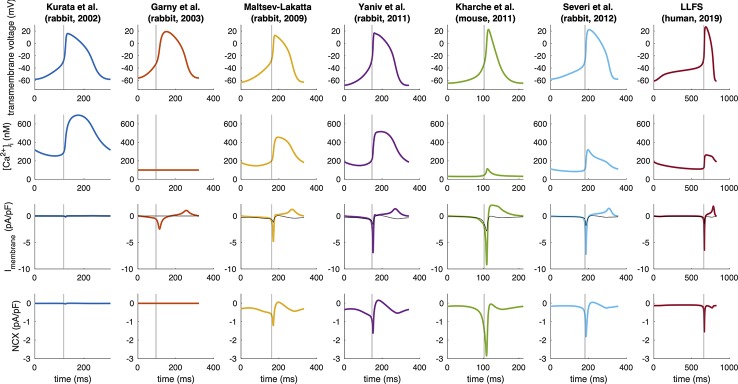
Spontaneous APs, Ca^2+^ transients, total membrane currents, and NCX in computational models of different species (compare **Figure 3**). Note the different time axes as a result of the markedly different spontaneous beating rates. The vertical gray line indicates the time of AP takeoff. For comparison, NCX is plotted in gray in the total membrane current panels.

**Figure 5** shows the time courses of V_m_, Ca^2+^_i_, total membrane current and NCX for several computational models of different species. Magnitude and temporal dynamics of NCX vary markedly between models and species. While NCX does not play a major role in the Garny et al. and Kurata et al. *rabbit* models, there is some early diastolic NCX in the Severi et al., Kharche et al. and Loewe–Lutz–Fabbri–Severi (LLFS) models, which becomes more pronounced only slightly before AP ignition. In the Yaniv et al. and Maltsev–Lakatta models in contrast, NCX is apparent markedly earlier. A current integral reflects the total charge transported across the membrane during a specific period of time. For a current normalized to the membrane capacitance (pA/pF), the integral is identical to the potential difference caused by the charge accumulated on the capacitive membrane due to the current (units: ms * pA/pF ≡ mV) is a measure of how much of the DD (in terms of mV) can be attributed to NCX. To quantify the role of NCX to DD in the models of different species, we computed the NCX integral during DD:

(2) $$\left|\overset{t_{takeoff}}{\underset{t_{MDP}}{\int }}I_{NaCa}\left(t\right)dt\right|,$$

where t_takeoff_ was defined as the first time step for which d^2^V_m_/dt^2^ exceeded 15% of the maximum d^2^V_m_/dt^2^ in the time interval between MDP and max(V_m_). The curvature based measure was inspired by the shape of the SN APs with the threshold empirically chosen to strike a balance between a detection early in the upstroke and robustness to minor increases of d^2^V_m_/dt^2^ during DD. **Figure 3** compares the NCX integral in relation to the total rise in V_m_ during DD and gives an idea about how much of the total DD can be attributed to NCX in each of the models across different species. Under the simplifying assumption of independent currents, almost the entire DD can be attributed to NCX in the Severi et al. rabbit and Kharche et al. mouse model. In the Yaniv et al., LLFS, and Maltsev–Lakatta models, NCX alone would cause more DD than the net ΔV_m_ observed, *i.e.*, outward currents partly compensate the NCX-induced depolarization whereas the role of NCX is almost negligible in the Garny et al. and Kurata et al. rabbit models.

**Figure 6:**
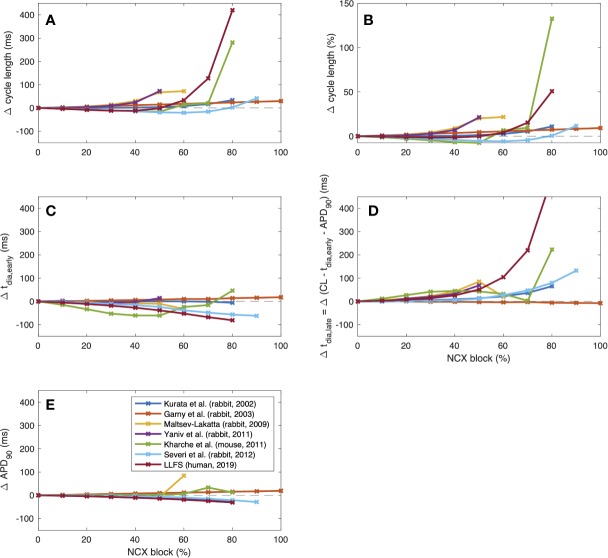
Change of CL [absolute **(A)** and relative **(B)**], t_dia,early_ **(C)**, t_dia,late_ **(D)** and APD **(E)** of computational models of different species (compare **Figure 4**) in response to gradual NCX block.

**Figure 7:**
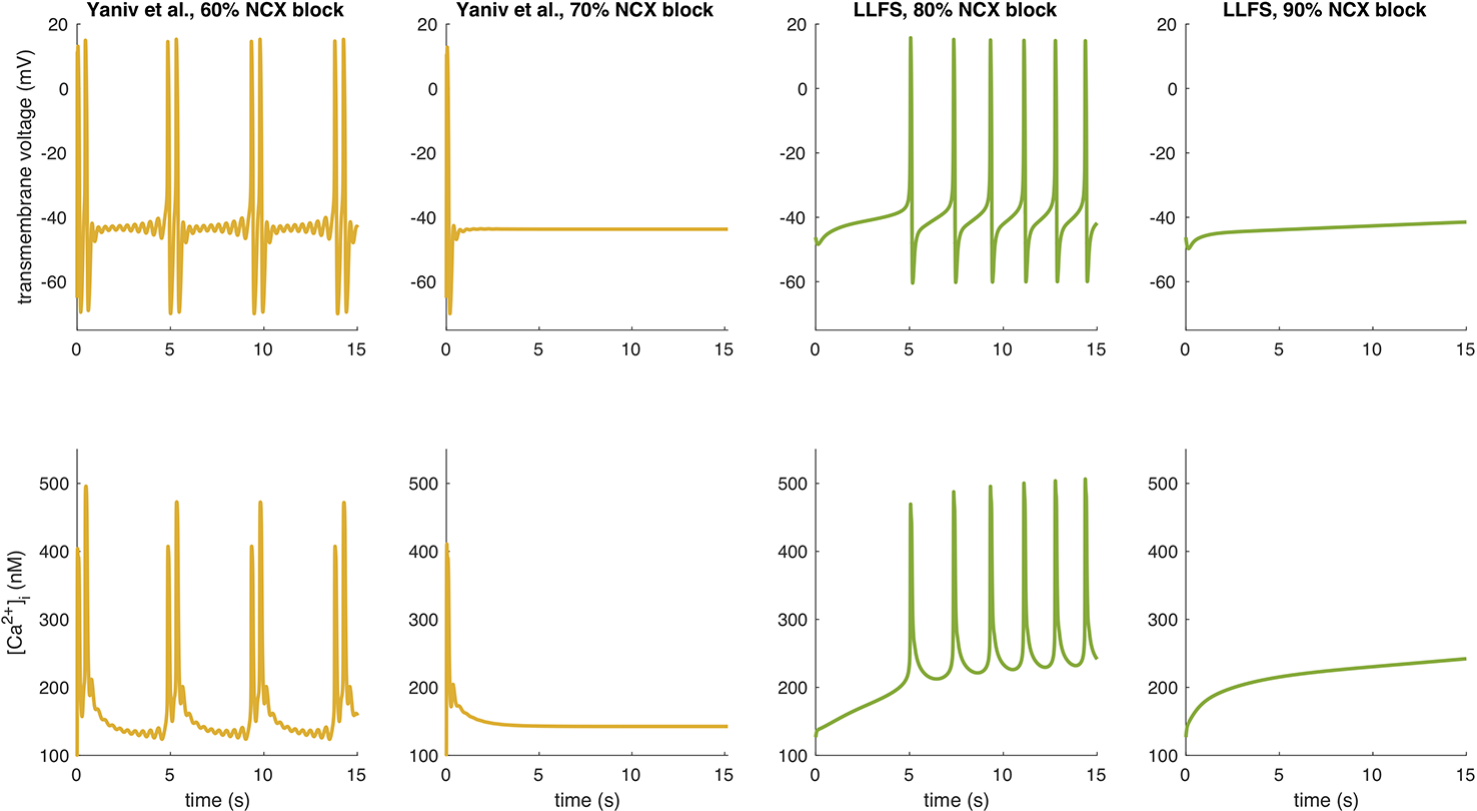
APs and Ca^2+^ transients of the Yaniv et al. and LLFS models for NCX block degrees slightly below and above the threshold for cessation of automaticity.

To further illustrate interspecies differences in the role of NCX in SN pacemaking, we evaluated how the models of different species respond to gradual block of NCX. **Figure 6** shows that the Garny et al. model exhibits an almost linear relation between NCX block and CL prolongation, however with a limited overall effect of +29.6 ms upon complete elimination of NCX. All other models required a certain amount of remaining NCX for reliable pacemaking (between 10% and 50%). In the Yaniv et al. model, 60% NCX block already terminated stable pacemaking. Instead, blocks of two subsequent APs followed by low amplitude V_m_ oscillations for several seconds were observed (compare **Figure 7**). The Yaniv et al. and Maltsev–Lakatta models showed a monotonic relation with CL prolongation for all degrees of block. In contrast, the Severi et al., Kharche et al., and LLFS models exhibited a biphasic relation with CL shortening up to a certain degree of block (80%, 50%, 50% block, respectively) and pronounced CL prolongation beyond (up to +420 ms in the LLFS model).

**Figure 8:**
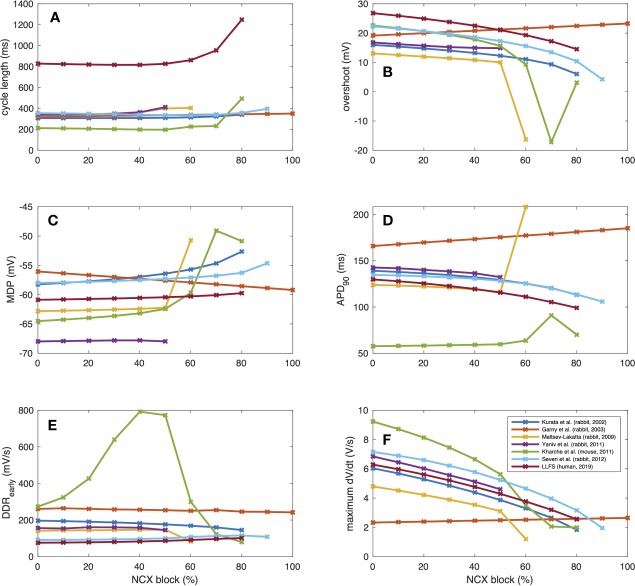
AP properties of computational models of different species (compare **Figure 3**) in response to gradual NCX block: CL **(A)**, overshoot **(B)**, MDP **(C)**, APD **(D)**, early DDR **(E)**, (dV_m_/dt)_max_ **(F)**.

To identify the drivers for these CL changes across species, we evaluated the effects on APD as well as early and late depolarization with the following metrics:

(3) $$\;\;\;\;\;\;\;\;\;\;\;\;\;\;\;\;\;\;\;t_{dia,early}=\;\frac{V_{takeoff}-MDP}{DDR_{early}}\;,\;\;\;\;\;\;\;\;\;\;\;t_{dia,late}=\;t_{takeoff}-t_{dia,early}\;,$$

where DDR_early_ was defined as a first order approximation of the DDR during the first 35 ms for the small animal models and the first 100 ms for the *human* LLFS model. APD shortening was responsible for the initial CL shortening in the Severi et al. rabbit and LLFS human models in combination with a reduction of t_dia,early_, which also affected the CL of the Kharche et al. model for degrees of block ≤70%. The superlinear CL prolongation for high degrees of block was mainly driven by changes in t_dia,late_, *i.e.*, changes that could not be attributed to APD, V_takeoff_, or changes in early DDR. Absolute values of CL, overshoot potential, MDP, APD_90_, DDR_early_, and dV_m_/dt_max_ upon NCX block of different degrees are shown in **Figure 8**. In addition to the effects included in the integral measures in **Figure 6**, a monotonic reduction of AP upstroke velocity could be observed in all but the Garny et al. rabbit model. As models of the same species (such as the Severi et al., Garny et al., and Yaniv et al. rabbit models presented here) sometimes exhibit different properties, it cannot be clearly pinned down which of the effects are truly species-dependent and which are simply model-dependent. Future studies should address this issue to identify which of the insights derived from animal experiments can be transferred to the human setting in which ways.

To study the biophysical identity of human SNCs, Chandler et al. (2009) performed an mRNA and protein expression analysis of human SNCs suggesting similar NCX1 in SN and atrial cells (Chandler et al., 2009). Based on their experimental findings, they derived one of the first computational models of human SNCs by adapting the atrial Courtemanche et al. (1998) model (Courtemanche et al., 1998) based on their experimental findings where NCX was scaled down by 26%. Allah et al. (2011) found rabbit SN NCX1 mRNA expression levels to be 78% of that of right atrial and 69% of that of left ventricular myocytes, respectively (Allah et al., 2011). Based on these two studies and I_f_ recordings in human SNCs, Verkerk et al. presented another early human SNC model (Verkerk et al., 2007; Verkerk et al., 2013). Compared to rabbit cells, their model of human SN cells had smaller absolute NCX, smaller absolute I_f,_ and a less pronounced Ca^2+^ transient. When comparing to the net diastolic membrane current, NCX was of similar magnitude between human and rabbit cells whereas I_f_ remained smaller in human cells.

The first human SNC model originating from a SNC model rather that an atrial model was presented by Fabbri et al. (2017). Their model is based on the Severi et al. rabbit model (Severi et al., 2012) and considered human data wherever available (Fabbri et al., 2017). The NCX formulation was adopted from the parent model and goes back to Kurata et al. (2002) (Kurata et al., 2002). As a result of the model parameter fitting to experimental data on the AP and Ca^2+^ transient level, the maximal NCX current was reduced by 53% (3.34 nA) compared to the parent model. Interestingly, NCX block experiments caused an increase in beating rate in the Fabbri et al. model. (50% block: 83 bpm [+12.2%]; 75% block: 93 bpm [+25.7%]) mediated by a synergistic acceleration of DD caused by elevated Ca^2+^_i_, shortening of APD and less negative maximum diastolic potential. Upon 90% NCX block, automaticity ceased. The *β*-adrenergic rate modulation experiments by Fabbri et al. did not include any direct effects on NCX but the SR Ca^2+^ pumping was modeled as a target of isoproterenol. The application of 1 µM isoproterenol yielded a 27.9% increase in beating rate. If only SR Ca^2+^ pumping was modeled as a target of isoproterenol, this increase was markedly attenuated and even slightly reversed with a remaining effect of −0.4%. Loewe et al. proposed the Loewe–Lutz–Fabbri–Severi model (LLFS) (Loewe et al., 2019b) as an extension of the Fabbri et al. model which considers, among other improvements, the dynamics and transients of the intracellular sodium and potassium concentrations in contrast to the previously fixed concentrations, thus further constraining the model physiologically by requiring homeostasis across time spans of minutes (Loewe et al., 2019a). In the LLFS model, the response to complete I_f_ block (CL +25.9%) was in accordance with the available experimental *human* data [+26% reported by Verkerk et al. (2007)] (Verkerk et al., 2007).

## Local Control of NCX

Several studies suggested that in cardiac muscle (Lipp et al., 1990) and other cells such as squid axon (Mullins and Requena, 1979), pancreatic acinar cells (Osipchuk et al., 1990), and smooth muscle (Stehno-Bittel and Sturek, 1992) ionic concentrations close to the intracellular surface of the plasma membrane may be different from those in the cytoplasm. These concentration gradients are thought to result from transient net fluxes across the surface membrane and may crucially influence the transmembrane ion channels. In atrial cells, which lack a well-developed t-tubule system, a non-uniform distribution of Ca^2+^ within the cell following release from the SR has been found and the results were interpreted in terms of distinct release sites under different control mechanisms (Lipp et al., 1990). Trafford et al. in rat ventricular myocytes also demonstrated the existence of the submembrane “area” by using the NCX current as a reporter and found that Ca^2+^_i_ changes near the surface membrane can be considerably larger than those averaged over the cell. The differences can be explained by a diffusion barrier between the release site and the bulk cytosol (Trafford et al., 1995).

In SNCs, the diastolic NCX is not only regulated by V_m_ but also by the LCRs, which in turn depend on local cross-talk including Ca^2+^-dependent functions of NCX, RyR, and SERCA (**Figure 1B**). This feedback mechanism between LCR and NCX can rapidly change NCX. It is known that RyRs are tightly organized in clusters, which are coupled with the L-type Ca^2+^ channels to form Ca^2+^ release units (CRU). RyRs are activated by an increase of local Ca^2+^_i_ resulting in Ca^2+^-induced Ca^2+^ release (CICR). Each CRU generates small, temporal Ca^2+^ release events, called Ca^2+^ sparks which have a radius of 1.5 µm (Stern et al., 2013; Hoang-Trong et al., 2015). Therefore, a LCR (4–12 µm) involves several sparks fired by neighboring CRUs *via* fire-diffuse-fire propagation (Maltsev et al., 2011a; Stern et al., 2014). Spark characteristics can be modulated by *β*-adrenergic response *via* variation of single CRU Ca^2+^ currents (I_spark_). The LCR controls SN chronotropy (acceleration and slowing) *via* changing the LCR size, amplitude, and number per cell surface area.

**Figure 9:**
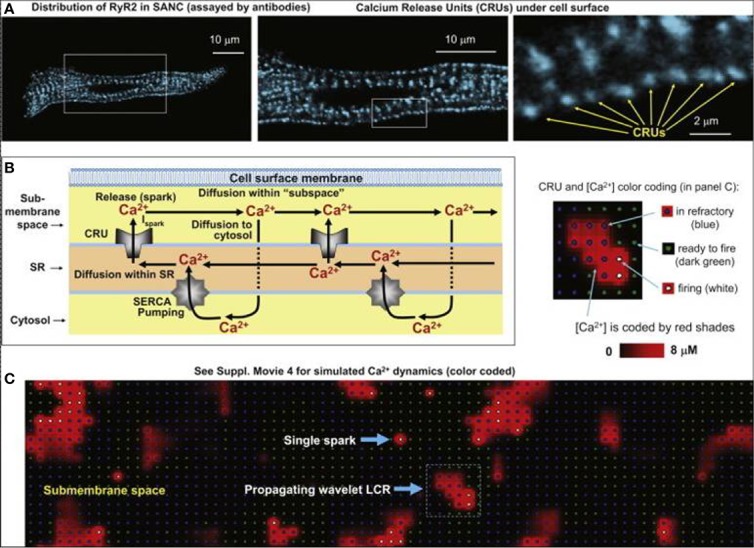
Development of the numerical model of local Ca^2+^ dynamics in rabbit SNCs: Ca^2+^ release units (CRUs) and Ca^2+^ cycling. RyR clusters, *i.e.*, CRUs, are visualized in confocal images of immuno-fluorescence in rabbit SNCs under cell surface membrane **(A)**. Schematic drawing of local Ca^2+^ cycling through different cell compartments (submembrane space, SR, and cytosol) **(B)**. Local Ca^2+^ fluxes are depicted with arrows, Ca^2+^ waves travel from left to right. **(C)** Instantaneous distributions of local [Ca^2+^] in the submembrane space predicted by our model: single Ca^2+^ spark and a wavelet LCRs (propagating from *left* to *right*). Inset illustrates the color-coding for CRU states and [Ca^2+^] (from (Maltsev et al., 2011a) with permission).

In 2011, Maltsev et al. demonstrated, by two-dimensional Ca^2+^ measurements on rabbit SNCs and *via* numerical modeling, that increasing the I_spark_ amplitude results in local synchronization of CRU firing yielding increasing LCR size, rhythmicity, and occurrence rate (Maltsev et al., 2011a). I_spark_ regulates both the time shift and the amplitude of the spontaneous release. Since the LCRs are directly coupled with NCX, this means that I_spark_ amplitude indirectly influences NCX during DD (**Figure 9**). In 2013, Maltsev et al. discovered a novel stabilization mechanism *via* local control of CICR (Maltsev et al., 2013). The Ca^2+^ released from the RyRs during DD is able to recruit the neighboring RyRs to release more Ca^2+^. This local recruitment depends on the extent of released Ca^2+^ and its diffusion to the neighboring RyRs. Since NCX extrudes Ca^2+^ from the vicinity of RyRs, it restrains CICR because the Ca^2+^ occurrence to diffuse and activate neighboring RyRs is reduced. The diastolic NCX increase is described by:

(4) $$\text{NCX}={\text{n}}_{\text{sparks}}\times {\text{NCX}}_{\text{spark}},$$

where n_sparks_ is the number of Ca^2+^ sparks and NCX_spark_ is the NCX current generated by one spark. This formula predicts that during NCX suppression the decreased NCX current (NCX_spark_) in turn increases the number of sparks (n_sparks_) since there is more Ca^2+^ available for diffusion and activation of additional RyRs. As the above formula predicts, the larger number of sparks tends to increase the NCX current, and finally, these opposite changes are able to largely compensate each other providing marginal change in the NCX. Stern et al. in 2014 provided three-dimensional SNC computational modeling including diffusion and buffering of Ca^2+^ in the cytosol and SR but omitted the submembrane space and inactivation of RyRs because of the lack of experimental validation data (Stern et al., 2014). Immunostaining experiments revealed that bridging RyR groups exist between large RyR clusters, organized in irregular networks. This architecture provides the basis for propagating LCR events. When a structure without bridges was incorporated in the model, the separated RyR clusters produced only isolated sparks, without LCRs and NCX activity. The model indicates that this hierarchical RyR clustering provides a pivotal adaptive mechanism and contributes to the *β*-adrenergic adaptation. Maltsev et al. (2017) characterized the complex spatiotemporal structure of LCRs in cardiac pacemaker cells (Maltsev et al., 2017). Using automatic classification of LCRs, they demonstrated that SR pumping controls LCRs and pacemaker rate *via* timely synchronized occurrence of LCRs creating a powerful ensemble signal to activate NCX. This also means that when SR pumping rate is low, the LCRs exert smaller amplitudes due to the lower SR Ca^2+^ load. However, the system compensates and maintains the NCX signal by increasing LCR size and duration. This mechanism ensures fail-safe pacemaker function within a wide range of pacing frequencies.

## Heterogeneity of SNCs

Functionally and electrophysiologically, the SN has a heterogeneous structure [as excellently reviewed by Boyett et al. (2000) and Lancaster et al. (2004)]. During normal cardiac activation, the spontaneous AP is initiated from the center, the leading pacemaker site of the SN, and then the AP is conducted through the periphery to the surrounding atrial muscle of the crista terminalis (Bleeker et al., 1980). In response to different interventions, the leading pacemaker site can shift to the peripheral region causing major importance in the pacemaker function. In the center the cells are described being smaller compared to those in the periphery and contain fewer myofilaments (Bleeker et al., 1980; Masson-Pevet et al., 1984; Boyett et al., 2000). The possible differences in the AP characteristics and the expression of ion channels were intensively studied, and the question whether the expression levels of proteins depend on the cell size of the SNCs remains equivocal (Honjo et al., 1996; Boyett et al., 1998; Lyashkov et al., 2007). The majority of the studies report that the expression of the Ca^2+^ regulatory proteins differs between regions of the SN, which results in heterogeneity of intracellular Ca^2+^ handling and pacemaker activity between cells across the nodal tissue. Musa et al. showed that the density of I_CaL_ was significantly (p < 0.001) correlated with cell capacitance in rabbit sinoatrial node, and larger cells showed greater density (Musa et al., 2002). The authors also performed immunocytochemical labeling of L-type Ca^2+^ channel, RYR2, and SERCA2, showing in significantly higher density in cells from the periphery compared to the center of the SN (Musa et al., 2002). In line with this, larger cells exhibited greater systolic Ca^2+^, diastolic Ca^2+^, Ca^2+^ transient amplitude, and spontaneous rate compared with smaller, probably central cells (Lancaster et al., 2004). Sarcolemmal Ca^2+^ ATPase and NCX have a lower activity in central cells, and the exchanger is responsible for a larger proportion of sarcolemmal Ca^2+^ extrusion in those cells compared with larger, peripheral cells (Lancaster et al., 2004). In 2007, Lyashkov et al. determined the density and distribution of cRyR, NCX1, and SERCA2 in SNCs of different sizes from the rabbit SN tissue (Lyashkov et al., 2007). They found that both bigger and smaller SN cells have identical AP-induced Ca^2+^ transients and spontaneous localized Ca^2+^ release characteristics (Lyashkov et al., 2007). They provided evidence on robust NCX1 as well as SERCA2 and cRyR cellular labeling densities in SNCs and they found that the entire sinoatrial node—including the Cx43 negative primary pacemaker area—exhibits positive labeling for NCX1, cRyR, and SERCA2 (Lyashkov et al., 2007). Furthermore, submembrane colocalization of NCX1 and cardiac RyR was shown, which exceeds the degree observed in atrial or ventricular cells (Lyashkov et al., 2007). However, Tellez et al. examined the molecular basis of the ion currents in rabbit SN with measuring the abundance of messenger RNAs coding for ion channels and regulatory proteins of the SN tissue. Ca^2+^ handling proteins abundance showed variability between tissues from different parts of the SN (Tellez et al., 2006). The abundance of NCX1 mRNA was significantly lower in the periphery of the SN compared with other regions. In the transcript profile, an apparent isoform switch from the atrial muscle to the center of the SN was found: RYR2 to RYR3, Na_v_1.5 to Na_v_1.1, Ca_v_1.2 to Ca_v_1.3 and K_v_1.4 to K_v_4.2 (Tellez et al., 2006).

Maltsev and Lakatta in 2013, by using a numerical model, explored large variation of ensembles of electrogenic molecules to identify the minimum set of proteins that confer a robust (i.e. fail-safe) and flexible (i.e. adaptation to a given vegetative stimulus) pacemaker function (Maltsev and Lakatta, 2013). The minimal model was found to contain: I_CaL_+ I_Kr_+ NCX+ Ca^2+^ clock. The further addition of I_CaT_ and I_f_ decreased flexibility but increased robustness of the system. Therefore the higher balance of flexibility and robustness contains five parameters: I_CaL_ + I_Kr_ + NCX + Ca^2+^ clock + I_f_. It is important to note that in the four parameter models, all model sets without NCX failed to generate rhythmic APs in at least one of the tests, confirming the fundamental importance of the exchanger in the pacemaking.

Monfredi et al. examined the major ionic currents in intercaval pacemaker cells (IPCs) obtained from rabbit heart and found marked electrophysiological heterogeneity in IPCs (Monfredi et al., 2018). Experiments and numerical simulations indicated that there is an IPC cell population with minimal or zero I_f,_ without correlation between I_f_ density and cell size. In the absence of I_f_, the diastolic NCX in response to Ca^2+^ release (*i.e.*, the Ca^2+^ clock) could drive the pacemaking. Wide ranges of I_CaL_ and I_K_ densities were found in these cells. Functional heterogeneity of SNCs was recently described by Kim et al. (2018). Their experiments performed in guinea pigs demonstrated three types of SNCs: rhythmically firing, dysrhythmically beating, and dormant cells exerting no spontaneous activation. LCRs were present in all three types; however in the dysrhythmic and dormant cells only smaller, stochastic LCRs without spatial and temporal synchronization were observed. *β*-adrenergic stimulation synchronized LCRs in all dysrhythmic cells and in the 1/3 of the dormant cells. Furthermore, the dormant cells developed automaticity in response to *β*-adrenergic stimulation. These results indicate partial or complete uncoupling between Ca^2+^ and M-clocks in the dysrhythmic and dormant cells.

## What is the Role of NCX in Pacemaking Under Resting Heart Rate?

When active in forward mode, NCX generates inward current and at the same time represents the most important pathway for Ca^2+^ extrusion to maintain the Ca^2+^ flux balance (Bers, 2001). Since these two functions are not separable, profound (but incomplete) NCX inhibition may always have secondary effects by altering the intracellular Ca^2+^ levels. Considering the published results and the nature of NCX inhibition, it could be that pharmacological NCX inhibition (partial for all available agents) or incomplete transgenic knockout mutants are not able to unequivocally pin down NCX's function in pacemaking. Theoretically, partial NCX suppression (acute pharmacological, or incomplete transgenic models) may lead to 3 different outcomes, characterized by the intracellular Ca^2+^ concentration since NCX is the major pathway for Ca^2+^ extrusion:

i) NCX inhibition does not increase Ca^2+^_i_ because of activation of other compensatory mechanisms (such as plasma membrane ATPase). In this fortunate case, all NCX inhibition is pure current inhibition with minimal secondary, indirect mechanisms through changes of Ca^2+^_i_. However, this scenario may have the lowest probability.
ii) NCX inhibition does not increase Ca^2+^_i_ because the (potentially small) uninhibited fraction is able to remove the Ca^2+^. This suggests a non-linear relationship between the degree of NCX inhibition and reduction of NCX function in terms of Ca^2+^ extrusion during the DD, making the interpretation difficult.
iii) NCX inhibition elevates Ca^2+^_i_. In this case, the Ca^2+^ gain will enhance the Ca^2+^-dependent inactivation of the I_CaL_, which may shorten the AP. However, also the AP threshold is expected to be higher, requiring more time to be reached. According to the ignition theory (Lyashkov et al., 2018), the decreased contribution of I_CaL_ also reduces the I_CaL_-LCR-NCX interactions. Furthermore, the Ca^2+^ increase may activate other Ca^2+^-dependent mechanisms such as I_K(Ca)_. These indirect mechanisms may counteract the effect of NCX inhibition.

Groenke et al. demonstrated that NCX1 KO mice show no spontaneous atrial depolarization (Groenke et al., 2013); only junctional escape rhythm was observed. The cells did not show spontaneous automaticity despite the presence of I_f_ and intact Ca^2+^ stores. Current mechanistic models of both human (Loewe et al., 2019a) and rabbit (Yaniv et al., 2011) SNCs also support no spontaneous automaticity when NCX function is completely absent. Considering pharmacological data, it can be firmly stated that different maneuvers aiming to decrease the intracellular Ca^2+^ level such as ryanodine, CPA, BAPTA in all cases lead to a decrease of SN frequency to varying extents. However, the kinetics of Ca^2+^ currents also changes due to these interventions. Therefore, taking together the published results, it is doubtless that if NCX is largely inhibited or completely absent, the normal Ca^2+^ flux balance is considerably impaired which per se may influence spontaneous automaticity. These secondary shifts of Ca^2+^ balance make the experimental evaluation of the total NCX contribution during DD more difficult. At the same time, the available experimental data with manipulation of Ca^2+^_i_, modifications of second messengers (such as cAMP, PKA, PDE, CaMKII), transgenic animals and numerical modeling clearly indicate the fundamental role of NCX in maintaining the resting heart rate.

## What is the Role of NCX in the Fight-or-Flight Response?

The *β*-adrenergic response involves the increase of intracellular cAMP and PKA, increases CaMKII activity and increases the intracellular Ca^2+^ load *via* phosphorylation of various channels (**Figure 1B**). A large body of evidence indicates that LCRs and therefore also NCX are under the influence and delicate control of several intracellular signaling molecules (such as CaMKII, PKA, cAMP, PDE), which are sensitive to *β*-adrenergic activation (see *The Role of Phosphorylation in SN Pacemaking and the Fight-or-Flight Response*). These results indicate a pivotal role of NCX not only in maintaining the basal SN automaticity but also in the sympathetic stimulus-mediated heart rate acceleration.

## Does the Reverse Mode of NCX Influence SN Pacemaking?

Currently, a transport-selective NCX inhibitor is not available. However, the KB-R7943 preferentially inhibits the reverse mode of NCX (Birinyi et al., 2005); it markedly influences I_CaL_ (Birinyi et al., 2005) making the interpretation difficult. The electrophysiological properties of the NCX predict functioning reverse mode when V_m_ is depolarized (more than 0 mV) and Na^+^_i_ is high. Experimental results as well as modeling data demonstrate that SN APs peak around 20 mV, which could be considered high enough to “switch on” reverse mode; however the time at depolarized levels is very short. During normal Na^+^_i_, mechanistic modeling predicts only marginal reverse mode at the beginning of the AP, thus the NCX current is mainly inward throughout the entire AP. The Yaniv et al. (Yaniv et al., 2013b) and LFSS model (Loewe et al., 2019a) predicted termination of beating when intracellular Na^+^_i_ is elevated, suggesting that this is not a way to augment reverse NCX during SN automaticity.

Maltsev and Lakatta in 2013 (Maltsev and Lakatta, 2013) reported a notable reverse mode of the NCX that was lacked in the previously published models. Numerical calculations revealed 2.45 pC of Ca^2+^ influx *via* reverse mode that is comparable with I_CaL_-mediated Ca^2+^ influx for one AP cycle. This means that reverse NCX contributes in “refueling” the Ca^2+^ clock almost equal to I_CaL_.

## Criticisms of CA^2+^-Theory

The role of Ca^2+^_i_ in the SN pacemaker function has been challenged from the beginning. However, the importance of intact Ca^2+^ handling in normal SN automaticity was accepted in a relatively short time. In contrast, the dominance of I_f_ over NCX and *vice versa* was a recurrent and often appearing question (Lakatta and DiFrancesco, 2009). Since the funny-current was the first proposed mechanism underlying pacemaking, it is hardly surprising that the Ca^2+^ theory was challenged many times experimentally.

### Ryanodine Effect

Serious criticisms have emerged regarding the frequency modulating effect of ryanodine. Authors often found a 15–30% frequency decrease after application of ryanodine which was attributed to the effect of indirectly reduced NCX activity. However, Bucchi and DiFrancesco in 2003 showed that the ryanodine mediated increase of CL is not the consequence of decreased DD slope but the positive shift of AP threshold (Bucchi et al., 2003). A further consistent finding was that ryanodine considerably reduces the frequency-increasing effect of *β*-adrenergic activation (Bucchi et al., 2003). The DiFrancesco group (Bucchi et al., 2003) demonstrated that ryanodine disrupts the normal signal transduction pathway of of β-adrenergic response, and the intracellularly used cAMP analogues were able to increase the frequency in the presence of 3 µM ryanodine to a similar extent as during control. The authors claim that the SR Ca^2+^ release may represent only a “safety mechanism” for the DD to reach the threshold level. Another paper of DiFrancesco group (Bucchi et al., 2007b) investigated “signatures” of rate changes caused by different pharmacological maneuvers. They found that agents modifying I_f_ such as ivabradine, isoproterenol, cAMP, and acetylcholine affect only the slope of DD while ryanodine-mediated bradycardia is caused by an increase of the take-off potential. They conclude that while other mechanisms could be important I_f_ appears to be the simplest and most direct contributor to rate modulation. While the reduction of pacing rate after ryanodine application is an unequivocal finding, the underlying mechanisms (change of take-off potential *vs.* slowed DDR) are a matter of debate. Vinogradova et al. showed that ryanodine has a marked effect on the DD by decreasing DDR, thus causing significant CL prolongation (Vinogradova et al., 2002). In another study, they also observed that in the presence of ryanodine, the ability of the cAMP analogue CPT-cAMP to increase the SNC beating rate was markedly reduced from ~35% (in control) to ~10% (Vinogradova et al., 2006). The different findings of this study and the previously mentioned study by Bucchi et al. (2003) could be partly due to different experimental approaches. DiFrancesco's group used clusters of SNCs with whole cell recordings of APs, while Lakatta's group used single SNCs with the perforated patch technique.

However, ryanodine may have a direct inhibitory effect on I_CaT_ channels, which would complicate the interpretation of such experimental data (Li et al., 1997).

Bucchi et al. and Vinogradova et al. (Vinogradova et al., 2002; Bucchi et al., 2003; Vinogradova et al., 2006) were not the only ones investigating the effects of RyR Ca^2+^ release suppression by ryanodine in the response of *β*-adrenergic stimulation. The influence of ryanodine on the modulation of the chronotropic effect of *β*-adrenoceptor stimulation has been extensively studied in a variety of different species. In guinea-pig SNCs exposed to 100 nmol/L isoprenaline, a decrease in firing rate and decrease in the amplitude of Ca^2+^ transients were observed after ryanodine application, supporting that ryanodine decreases the positive chronotropic effect of isoprenaline (Rigg et al., 2000). In isolated mouse SNCs, in the presence of ryanodine, the chronotropic effect of *β*-adrenoceptor stimulation was entirely suppressed and a reduction of spontaneous AP frequency was observed after isoprenaline application (Wu et al., 2009). Consistent with this finding, Joung et al. showed that the isoprenaline dose-dependent increase of heart rate was suppressed by ryanodine infusion and SR Ca^2+^ depletion with ryanodine prevented isoproterenol-induced LDCaE and blunted sinus rate acceleration in canine right atrium (Joung et al., 2009).

It is important to note that the ryanodine effect highly depends on the concentration and experiment time (Rousseau et al., 1987; Smith et al., 1988; Buck et al., 1992; Zimanyi et al., 1992; Fill and Copello, 2002). In low doses, it locks the RyR in a subconductance open state (Buck et al., 1992). Before the SR depletion, the Ca^2+^ flux increases the firing rate indicating the direct role of Ca^2+^ and NCX in pacemaking (Rubenstein and Lipsius, 1989). The AP ignition model (Lyashkov et al., 2018) predicts that ryanodine substantially depolarizes the ignition potential and prolongs both CL and time-to-ignition, but does not significantly affect the MDP. Additional simulations with Ca^2+^ dynamics showed that Ca^2+^ release continues in the presence of SR Ca^2+^ leak (the leaky SR model mimics ryanodine-dependent lock of RyRs in the subconductance state) but it becomes persistent. In this case, the diffusional resistance between the network and junctional SR limits intra-SR flux and preserves the Ca^2+^-load of the network SR. This maintains SR Ca^2+^ load and drives persistent release flux (Lyashkov et al., 2018).

### Ca^2+^-Dependence of I_f_

A possible Ca^2+^-dependence of the funny current would raise serious questions since the maneuvers to alter Ca^2+^_i_ levels were generally considered to influence the SR-NCX axis only, without interfering with I_f_. Hagiwara and Irisawa (1989) demonstrated Ca^2+^ dependent changes of I_f_ (Hagiwara and Irisawa, 1989). Later inside-out patch clamp experiments revealed that Ca^2+^ ions do not directly influence the I_f_ current (Zaza et al., 1991). However, a Ca^2+^-activated adenylate-cyclase isoform was found in SNCs (Mattick et al., 2007), indicating that a Ca^2+^ rise after NCX inhibition will induce augmented funny current. This effect is also proposed to counterbalance the effect of NCX inhibition on the CL. Furthermore, it may be another link between Ca^2+^ homeostasis and V_m_. The actual value of the MDP is mainly defined by the potassium conductance during repolarization. Therefore, potassium channels indirectly influence automaticity. The small conductance Ca^2+^ activated potassium current may provide a direct link between Ca^2+^ handling and repolarization. Even though no effect was found in ventricular myocardium (Nagy et al., 2009), a possible contribution for pacemaking was reported (Torrente et al., 2017; Saeki et al., 2019).

### Intracellular Buffering

The forward mode of the NCX extrudes Ca^2+^ from the intracellular space while generating inward current that contributes to DD. Since the primary driver of NCX during DD is the Ca^2+^ concentration, buffering of intracellular Ca^2+^ affects the role of NCX and potentially attenuates or terminates spontaneous automaticity. Several authors challenged the role of SR release in setting the actual frequency of SN pacemaking by application of the rapid Ca^2+^ buffer BAPTA. The results are summarized in **Table 2**. The data clearly demonstrate the significant role of Ca^2+^_i_ in the SN pacemaking, *i.e.*, the function of the inward NCX during DD. A contradictory result was published by Himeno et al., where unaffected AP firing rate was demonstrated in the presence of high Ca^2+^_i_ buffering, thus challenging the Ca^2+^ hypothesis (Himeno et al., 2011). Their results were later interpreted by the Lakatta group (Maltsev et al., 2011b) as methodological problems, *i.e.* incomplete seal resistance. When rabbit cells were loaded with the caged Ca^2+^ buffer NP-EGTA, the firing rate was slow and dysrhythmic with low SR Ca^2+^ levels. Rapid photolysis induced Ca^2+^ increase, in turn, markedly increased the frequency (by about 50%), accelerated the DD, augmented the LCRs, and reduced the CL variability (Yaniv et al., 2011).

**Table 2:** Effects of BAPTA on spontaneous firing rate of SNCs.

Species	Preparation	Concentration	Period(s) of application	Control (bpm)	After drug (bpm)	Rate reduction (%)	Reference
Toad	Single sinus venosus cells	10 μM BAPTA	5 and 8 min	31	15	51.6	Ju and Allen et al.
Rabbit	Single SNc	5 μM BAPTA	7–10 min	149	69	53.7	Bogdanov et al.
Rabbit	Cultured SNc	25 μM BAPTA	10 min	94	65	30.9	Li et al.
Rabbit	Single SNc	5 μM BAPTA	5 min	174	80	54	Vinogradova et al.
Rabbit	Single SNc	25 μM BAPTA	25 min	210	120	43	Lyashkov et al.
Rabbit	Single SNc	25 μM BAPTA	30 min			87	Younnes et al.
Guinea pig	Single SNc	10 μM BAPTA	3 min			100%	Sanders et al.
Guinea pig	Single SNc	10 mM BAPTA	30 s	153	141	7.8%	Himeno et al.

### Pharmacological Problems

Important pharmacological problems arose regarding I_f_ and NCX inhibitors. The contribution of I_f_ was traditionally investigated using caesium or ivabradine. The heart rate lowering effects of these compounds varied within a large range (7–31%) and even 20 mM caesium was unable to terminate spontaneous SN beating (Sohn and Vassalle, 1995). Therefore, the pacemaking role of I_f_ was regularly challenged by considering its I–V relationship (small current in the DD range), slow activation kinetics, and moderate effects of inhibitors (DiFrancesco, 1995; Vassalle, 1995; Bucchi et al., 2007a; Lakatta and DiFrancesco, 2009). However, both CsCl_2_ and ivabradine cause incomplete and voltage-dependent block. A further issue with ivabradine is the significant I_Kr_ block even at the widely used concentration of 3 μM (Koncz et al., 2011). Similar situations can be found with selective NCX inhibitors. Both ORM-10103 and ORM-10962 however, exert no influence on other currents and partially inhibit NCX, may also depend on Ca^2+^. As discussed above (*What is the Role of NCX in Pacemaking Under Resting Heart Rate*)*?*, increased Ca^2+^ limits the effect of NCX inhibition *via* secondary effects and covers the exact role of NCX during DD. Taking together these results, it seems likely that complete pharmacological inhibition of I_f_ or NCX is currently not feasible using the available compounds, impeding the identification of the role of NCX and I_f_ in SN pacemaking.

An indirect experiment was also published in 2015 by Szepesi et al. (2015) using 10 µM ORM-10103 as a selective NCX inhibitor (Jost et al., 2013) in Langendorff perfused rat hearts. The results exerted identical R-R intervals after application of selective NCX blockade.

In 2008, Farkas et al. investigated the possible antiarrhythmic effect of selective NCX inhibition against dofetilide-induced Torsades de pointes arrhythmia by using 1 µM SEA0400, a nonselective NCX inhibitor in rabbit and rat Langendorff-perfused hearts (Farkas et al., 2008). However, the measured R-R intervals indicated no effect on sinus function of NCX inhibition in both species. In contrast, by using the most selective available NCX inhibitor ORM-10962, Kohajda et al. showed a tendency to decrease the heart rate in guinea pig hearts *in vivo*, however within the experimental variance (Kohajda et al., 2016). 1 µM ORM-10962 exerted statistically significant, however marginal (8%) decrease in rabbit atrial tissue, while Ca^2+^_i_ was significantly increased in SNCs (Kohajda et al., 2020).

## Applications

Besides its fundamental role for pacemaking under physiological conditions, various applications of engineered NCX and potential roles in pathogenesis have been put forward. Silva and Rudy (2003) numerically replicated biological pacemakers derived from guinea pig ventricular cells (Silva and Rudy, 2003). Suppression of I_K1_ by 81% elicited rhythmic spontaneous depolarizations at a CL of 594 ms caused by NCX as the main pacemaking current. The role of NCX was underlined by a bifurcation analysis of biological pacemaking driven by an unstable equilibrium point *via* a saddle-node bifurcation by Kurata et al. (2005) using a modified Priebe-Beuckelmann model in which NCX was identified as the primary pacemaker current (Kurata et al., 2005). However, they found that NCX is not necessarily required for rhythmic spontaneous depolarizations and that equilibrium point instability as a prerequisite for stable pacemaking is caused by I_CaL_ but not NCX. Jones et al. (2012) identified NCX as one of the key determinants of pulmonary vein automaticity, *i.e.* pacemaking in non-SN cells, which is considered as one of the most important triggers initiating atrial fibrillation (Jones et al., 2012). Youm et al. (2006) modeled pacemaker activity in mouse small intestine, *i.e.*, noncardiac, cells and found a role of NCX for automaticity in these cells as well (Youm et al., 2006).

Loewe et al. investigated the effect of extracellular Ca^2+^ concentration changes on SN pacemaking in the LLFS computational model motivated by the observation that the heart rate of dialysis patients, who experience marked shifts of extracellular ion concentrations, drops to very low values before they suffer from sudden cardiac death with an unexplained high incidence (Loewe et al., 2019a). They found that Ca^2+^ changes markedly affected the beating rate (46 bpm/mM ionized Ca^2+^ without autonomic control). While this pronounced bradycardic effect of hypocalcemia was mediated primarily by I_CaL_, NCX was subsequently attenuated due to the reduction of Ca^2+^_i_. Wolf et al. (2013) proposed a computational model of ankyrin-B syndrome SNCs in which alterations of I_CaL_ and NCX as well as I_NaK_ due to ankyrin-B dysfunction increase variability in SN automaticity (Wolf et al., 2013). Choudhury et al. (2018) overexpressed NCX1 in bradycardic rat subsidiary atrial pacemaker tissue and found that it did not accelerate the rate of spontaneous depolarizations in contrast to overexpression of TBX18, which increased rate as well as stability and rescued isoproterenol response (Choudhury et al., 2018).

## Concluding Remarks

A large body of experimental evidence indicates and mechanistic *in silico* modeling supports the crucial role of NCX in SN automaticity both under normal heart rate as well as during *β*-adrenergic stimulation. However, SN automaticity probably could be one of the areas of experimental cardiology where concise hypotheses have been derived from computational modeling that call for experimental testing which is not yet on the horizon, especially in context of NCX function. The complex effects of drugs modulating the Ca^2+^ handling (CPA, ryanodine, isoproterenol), the shortcomings of transgenic animal models, and the nonselectivity and/or indirect effects of parallel Ca^2+^ increase of selective inhibitors indicate a clear demand of further experimental research to fully reveal the role of NCX in SN pacemaking.

## Author Contributions

AL—preparing the manuscript: in silico mechanistic modeling, preparing the figures. AV—supervising the study. NN—preparing the manuscript: experimental results of the NCX function in SN. ZK, NT—searching of relevant publications, preparing subchapters of the manuscript.

## Funding

We declare that all sources of funding received is submitted.

## Conflict of Interest

The authors declare that the research was conducted in the absence of any commercial or financial relationships that could be construed as a potential conflict of interest.

## Abbreviations

AP, action potential; APD, action potential duration; ATP, adenosine triphosphate; Ca^2+^/CA, Ca^2+^-cation antiporter; Ca^2+^_i_, intracellular Ca^2+^ concentration; CaM, calmodulin; CaMKII, Ca^2+^/calmodulin-dependent protein kinase; cAMP, cyclic adenosine monophosphate; CICR, Ca^2+^-induced Ca^2+^ release; CL, cycle length; CRU, Ca^2+^- release unit; DD, diastolic depolarization; DDR, diastolic depolarization rate; EC50, half maximal effective concentration; E_NCX_, NCX equilibrium potential; GPCR, G-protein coupled receptor; hESC-CM, human embryonic stem cell-derived cardiomyocyte; IC50, half maximal inhibitory concentration; I_CaL_, L-type Ca^2+^-current; I_CaT_, T-type Ca^2+^-current; I_f_, funny-current; I_K1_, inward rectifier potassium current; I_K(Ca)_: small-conductance Ca^2+^-activated K^+^ current; I_Kr_, rapid delayed rectifier potassium current; I_Ks_, slow delayed rectifier potassium current; I_to_, transient outward potassium current; LCR, local Ca^2+^ releases; LDCaE, late diastolic Ca^2+^ elevation; LLFS model, Loewe–Lutz–Fabbri–Severi model; M-clock, membrane clock; MDP, maximal diastolic potential; Na^+^_i_, intracellular Na^+^ concentration; NCX, Na^+^/Ca^2+^ exchanger; NO, nitrogen-monoxide; PDE, phosphodiesterase; PKA, protein kinase A; PKC, protein kinase C; PLB, phospholamban; PP1, protein phosphatase 1; PP2A, protein phosphatase 2; RyR, ryanodine receptor; SERCA, sarcoplasmic reticulum Ca^2+^ ATPase; SN, sinus node; SNC, sinus node cell; SR, sarcoplasmic reticulum; V_m_, transmembrane potential.
